# Multi-Parametric Analysis and Modeling of Relationships between Mitochondrial Morphology and Apoptosis

**DOI:** 10.1371/journal.pone.0028694

**Published:** 2012-01-17

**Authors:** Yara Reis, Marti Bernardo-Faura, Daniela Richter, Thomas Wolf, Benedikt Brors, Anne Hamacher-Brady, Roland Eils, Nathan R. Brady

**Affiliations:** 1 Division of Theoretical Bioinformatics, German Cancer Research Center and Institute of Pharmacy and Molecular Biotechnology, Bioquant, University of Heidelberg, Heidelberg, Germany; 2 Systems Biology of Cell Death Mechanisms, German Cancer Research Center and Bioquant, Heidelberg, Germany; 3 Department of Surgery, Medical Faculty, University of Heidelberg, Heidelberg, Germany; 4 Institute of Cell and Molecular Pathology, Hannover Medical School, Hannover, Germany; Boston University, United States of America

## Abstract

Mitochondria exist as a network of interconnected organelles undergoing constant fission and fusion. Current approaches to study mitochondrial morphology are limited by low data sampling coupled with manual identification and classification of complex morphological phenotypes. Here we propose an integrated mechanistic and data-driven modeling approach to analyze heterogeneous, quantified datasets and infer relations between mitochondrial morphology and apoptotic events. We initially performed high-content, multi-parametric measurements of mitochondrial morphological, apoptotic, and energetic states by high-resolution imaging of human breast carcinoma MCF-7 cells. Subsequently, decision tree-based analysis was used to automatically classify networked, fragmented, and swollen mitochondrial subpopulations, at the single-cell level and within cell populations. Our results revealed subtle but significant differences in morphology class distributions in response to various apoptotic stimuli. Furthermore, key mitochondrial functional parameters including mitochondrial membrane potential and Bax activation, were measured under matched conditions. Data-driven fuzzy logic modeling was used to explore the non-linear relationships between mitochondrial morphology and apoptotic signaling, combining morphological and functional data as a single model. Modeling results are in accordance with previous studies, where Bax regulates mitochondrial fragmentation, and mitochondrial morphology influences mitochondrial membrane potential. In summary, we established and validated a platform for mitochondrial morphological and functional analysis that can be readily extended with additional datasets. We further discuss the benefits of a flexible systematic approach for elucidating specific and general relationships between mitochondrial morphology and apoptosis.

## Introduction

Mitochondria exist as a network of interconnected organelles and are responsible for generating the majority of ATP essential for cellular biochemistry. In response to specific stress stimuli, mitochondria participate in apoptosis via mitochondrial outer membrane permeabilization (MOMP), which results in the release of pro-apoptotic proteins to the cytosol [1], [2].

The number and morphology of mitochondria within a cell are a function of regulated rates of fusion and fission events [3]. Mitochondria display a complex architecture that varies from highly interconnected networks [4], to precisely structured individual units [5]. MOMP is not only regulated by interactions between pro- and anti-apoptotic Bcl-2 members, but also by a family of GTPases which control mitochondrial morphology (for review see, [6]). During the early stages of apoptotic cell death, network fragmentation and cristae remodeling are widely reported [7], [8], [9], [10], [11]. However, the relationship between morphology and apoptosis signaling remains unresolved, and can appear paradoxical. For example, pro-apoptotic Bax can promote mitochondrial fusion [12] and fragmentation may be preceded by increased fusion [13]. Furthermore, a pre-fragmented state confers protection by limiting mitochondrion-to-mitochondrion apoptotic signaling [10].

The overall goal of this study was to quantitatively investigate the relationship between mitochondrial morphology and programmed cell death. Therefore, we performed high-content measurements of mitochondrial morphologies, and apoptotic and energetic states in MCF-7 breast cancer cells, under control and drug-induced apoptotic conditions. We analyzed large sample populations as cell-to-cell heterogeneity in phenotypic responses is a critical source of biologically relevant information [14].

CellProfiler [15], [16] was used to perform automated image segmentation and feature extraction, generating rich parameter sets. From these sets we built a Random Forest (RF) classifier [17] using a supervised classification model, that was able to distinguish between networked, fragmented and swollen mitochondrial states, at the level of a single cell and within populations of cells. Measurements of critical MOMP parameters, i.e. Bax activation, mitochondrial membrane potential (ΔΨ_m_) and mitochondrial membrane depolarization were performed under matched conditions. To explore the relationships between morphological and functional parameters, these heterogeneous high-content datasets were integrated using data-driven fuzzy logic (FL) modeling. Our results suggest that mitochondrial morphological states are not linearly related to either Bax or ΔΨ_m_. Instead, FL modeling proposes a hierarchy of non-linear interactions between Bax, morphology, and ΔΨ_m_.

## Methods

### Cell culture and the induction of apoptosis

Human breast carcinoma MCF-7 cells (Cell Line Services; Heidelberg Germany) were cultured in DMEM (Invitrogen) supplemented with 10% FBS (Invitrogen), 1% penicillin/streptomycin (Invitrogen), 1% Glutamax (Invitrogen) and 1% nonessential amino acids (PAA laboratories) in a 37°C, 5% CO_2_ incubator. Cells were seeded overnight (5×10^5^ cells per well) and treated with the following compounds: C-6 ceramide (300 µM; Biozol), CCCP (20 µM; Calbiochem), TNFα (43 ng/mL; BASF), TRAIL (20 ng/mL; R&D Systems), thapsigargin (1 µM; Calbiochem), camptothecin (2 µM; BioVision), and oligomycin (10 µM; Sigma). Drug stocks were prepared according to manufacturer instructions. Drugs were diluted in balanced salt solution (BSS; Krebs-Henseleit Solution -in mM: 110 NaCl, 4.7 KCl, 1.2 KH2PO4, 1.25 MgSO4, 1.2 CaCl2, 25 NaHCO3, 15 glucose, 20 HEPES, pH 7.4) before application and incubated for 6 hours prior to all measurements.

### Expression plasmids and transfection

Plasmids encoding Mito-GFP (fusion of the localization tag of cytochrome *c* oxidase IV and GFP) [18] and GFP-Bax [19] were transfected into MCF-7 cells using Effectene (Qiagen) and positive clones were selected using neomycin (G418, 1 mg/mL; Carl Roth GmbH). Stable cell lines were generated from single colonies in order to minimize genetic background.

### Imaging procedures

All images were acquired using a wide-field DeltaVision RT (DVRT) deconvolution microscope.

#### Mitochondrial morphology

MCF-7 cells stably expressing Mito-GFP were seeded overnight (5×10^5^ cells per well) in an 8-well imaging µ-slide (ibidi) and treated with apoptotic drugs. Nuclei were stained with Hoechst (100 ng/mL; Sigma) for 1 minute prior to imaging. Live cells were imaged using a 63× oil objective (NA 1.40) and Z-stacks with 0.22 µm step sizes were collected and subsequently deconvolved using the bundled softWoRx software. The middle slice of the Z-stack was most representative of cellular mitochondrial content under all conditions, and was chosen for following analysis.

#### Mitochondrial membrane potential (ΔΨ_m_)

After respective drug treatments, MCF-7 wild-type (wt) cells were incubated with tetramethyl rhodamine methyl-ester (TMRM, 25 nM; Invitrogen) for 25 minutes at 37°C. Imaging was performed using a 40× air objective (NA 1.20). Sequential images of a single focal plane were acquired every second, over a period of 5 minutes. Exposure times were identical for each condition. For inhibition of the mitochondrial permeability transition pore (MPTP), MCF-7 wt cells were incubated in cyclosporine A (CsA, 5 µM; Calbiochem) for 30 minutes at 37°C or pre-treated with Bongkrekic acid (BA, 50 µM; Santa Cruz Biotechnology) for 1 hour at 37°C.

#### Bax activation

MCF-7 cells stably expressing GFP-Bax were incubated for 6 hours with the respective compounds and nuclei were stained with Hoechst (100 ng/mL; Sigma) before imaging (40× air objective, NA 1.20). 10 Z-stacks were acquired per condition and Z-projections (max) were preformed prior to analysis. 3D rendering was performed for representative image.

### Feature extraction of mitochondrial morphology

Mitochondrial morphology analysis was performed with CellProfiler software by combining available modules and submodules (www.cellprofiler.org), and configured to automatically (i) perform image preprocessing, (ii) segment and identify objects within the image (iii) and measure a selection of mitochondria and cell features. A detailed description of the CellProfiler pipeline and extracted features is available in Information S1 and Figure S1.

### Supervised classification (Random Forest)

The exported features were analyzed using a Random Forest (RF) model [17], which performed multidimensional data exploration and supervised machine learning-based image classification. The RF method is an ensemble classifier that consists of a family of decision trees. Each tree is constructed using a bootstrap sample of the data. The percentage of trees voting for a specific class is referred to as the RF score. Thus, the RF predictor assigns a degree of belonging between 0% and 100% to each class (networked/fragmented/swollen = N/F/S) per cell. At each iteration of the RF construction, the data not being in the training subsample (out of bag data) is used to estimate the error rate. The mean error estimation over all iterations is referred to as the out of bag (OOB) error. Peak importance is estimated by the mean decrease in accuracy (MDA). This score is the increase in OOB error when the OOB data for that peak is permuted while all others are left unchanged. A specific class can be assigned by taking in consideration only the class with the majority of the RF votes. However, this was only used for the 10-times-10-fold cross validation and validation purposes (comparison of the classifier with manual classification). All other data analysis steps used “raw” percentages given by the RF score, i.e. plotted results correspond to the mean value of each class assigned per cell (N/F/S) and reflect mitochondrial population distributions under a specific treatment.

### Mitochondrial membrane potential (ΔΨ_m_)

The release kinetics of the TMRM dye is here reported by the standard deviation (StDev) of the signal intensity from individual cells. Under normal conditions, mitochondrial TMRM is highly localized (high StDev) and upon ΔΨ_m_ loss, redistribution of the dye throughout the cell occurs and both total signal intensity and StDev decreases per cell [20]. From the StDev curves plotted for each condition, three parameters were extracted by using an automated MATLAB script: (i) t_1/2__decay: time for the signal-StDev to reach half of its initial value; (ii) Y_spread: total signal-StDev decrease over time; (iii) MAX: initial signal-StDev maximum value. The median of the first and last 10 points of each data set were used to calculate the maximum and minimum intensity. The t_1/2__decay is defined as the time point at which the StDev of the signal reaches half of its initial starting value (see Equation 1).
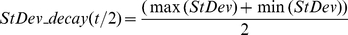



#### Equation 1

Definition of StDev value used in MATLAB script to extract t_1/2__decay parameter from StDev curves of TMRM signal.

### Fuzzy logic modeling

The fuzzy logic (FL) toolbox (MATLAB R2009a) was used to establish a modeling pipeline to perform exhaustive searches for relative correlations between measured events. Single input-single output (SISO) FL models were assembled using the Sugeno inference method. As a parameter reduction strategy, input membership functions (MF) were fixed to Gaussian functions, and thereby the number of input parameters was excluded from the model training. In FL, a Gaussian function has the form shown in equation 2, where the height of the peak is fixed to 1, i.e. the maximum degree of belonging to a fuzzy set (degree of membership, DOM).
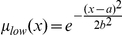



#### Equation 2

Gaussian equation as a membership function to establish the degree of membership (DOM) of a measurement 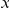 to the set *low*.

As output, linear MFs were chosen, and thus their stepwise combination allowed for the approximation of nonlinearity upon simulation.

In a FL system, the number of rules constitutes a free parameter, which we eliminated by using a fix number of rules. This number was the total of possible combinations of input MFs. Hence, this allowed the representation of all possible input-combination, while parameter fitting extracted from the data the degree to which this was happening in the specific measurement.

Training of the model was performed using a hybrid algorithm combining back propagation and iterative least-squares procedure [21]. Simulations of the SISO FL models were run using Simulink and root mean square errors (RMSE) were calculated. For the final step of the exhaustive search we selected the models with least-error. A detailed description of our FL modeling pipeline is available in Information S1 and Figure S2.

### Data analysis and statistics

Data is given as mean ± standard error of the mean (s.e.m). Statistical significance of differences was determined using a two-tailed Student's t-test. P values≤0.05 were considered to be statistically significant.

## Results

### Detection of mitochondrial morphology states by high-resolution imaging

**Figure 1:**
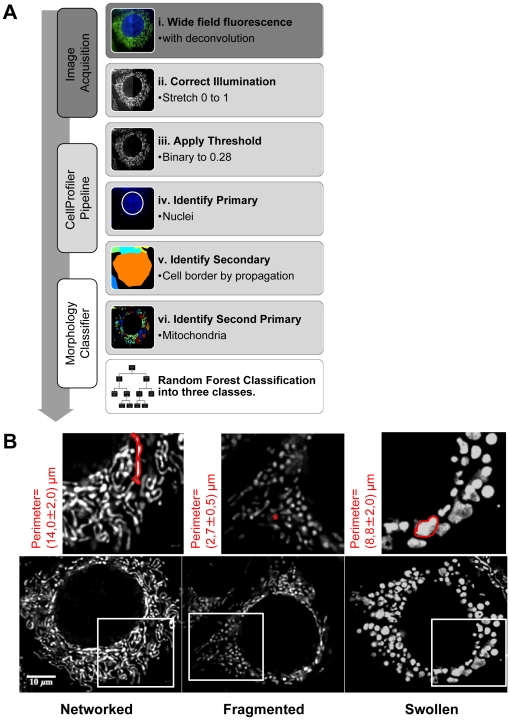
Mitochondrial morphology classification. A) Method pipeline - Principal modules: 1- Image acquisition; 2 - CellProfiler analysis; 3- Random Forest (RF) classification. MCF-7 cells stably expressing Mito-GFP were submitted to different apoptotic drugs for 6 hours at 37°C. Microscopic images were randomly acquired with a DeltaVision microscope and deconvolved: (i) shows half non-deconvolved (left) and half deconvolved (right) cell. Images were loaded into the following CellProfiler pipeline: (ii) illumination correction; (iii) threshold application; (iv) primary object (cell) identification from based on Hoeschst-labeled nuclei; (v) identification of cell borders; (vi) segmentation of individual mitochondria. Finally, 69 relative features were extracted and exported to build a Random Forest (RF) tree classifier. B) Cell-based classification - MCF-7 cells stably expressing Mito-GFP were incubated 6 hours under controlled conditions to induce networked (FM), fragmented (ceramide, 300 µM) and swollen (CCCP, 20 µM) mitochondria. Average perimeter values (in red) were measured from 10 mitochondria present in the zoomed region. Representative images correspond to the middle slice from 3D stacks.

Human MCF-7 breast cancer cells stably expressing mitochondrial targeted GFP (Mito-GFP) were imaged by high-resolution, widefield-fluorescence microscopy. All images were submitted to the workflow described in Figure 1A. Importantly, images were first deconvolved using a constrained iterative algorithm (Figure 1Ai) to increase the classification accuracy (92% accuracy for deconvolved vs. 65% accuracy for non-deconvolved; data not shown). Initial datasets were generated from putative conditions with enriched networked, fragmented and swollen phenotypes (Figure 1B). Networked states were obtained under full medium (FM) conditions. Fragmentation was induced by the pro-apoptotic lipid second messenger ceramide [22] and swelling was induced using the mitochondrial uncoupler CCCP (carbonyl cyanide m-chlorophenylhydrazone) [23].

Example images representing these three classes were initially characterized by manual classification of mitochondrial perimeters (Figure 1B). Perimeter size was greatest for networked mitochondria (14.0±2.0 µm), followed by swollen (8.8±2.0 µm) and fragmented (2.7±0.5 µm). Nevertheless, initial control perturbations revealed a high degree of perimeters variation within intracellular mitochondrial populations and among cell populations. Therefore, in order to analyze a significant amount of cells and exhaustively measure mitochondrial morphology states, we utilized the open source CellProfiler image analysis software [15], [16]. Our analytical pipeline comprised segmentation of individual nuclei, inference of cell boundaries, segmentation of mitochondria within assigned cells and feature extraction (Figure 1A). Parameter sets included mitochondrial size (e.g. area/volume), number (e.g. average per cell) and distribution within the cell, for a total of 69 features per cell (Tables S1, S2 and S3), which were exported to a MySQL database. All extracted features were the basis for building the mitochondrial morphology classifier algorithm.

### Machine learning based classification of mitochondrial morphology

**Figure 2:**
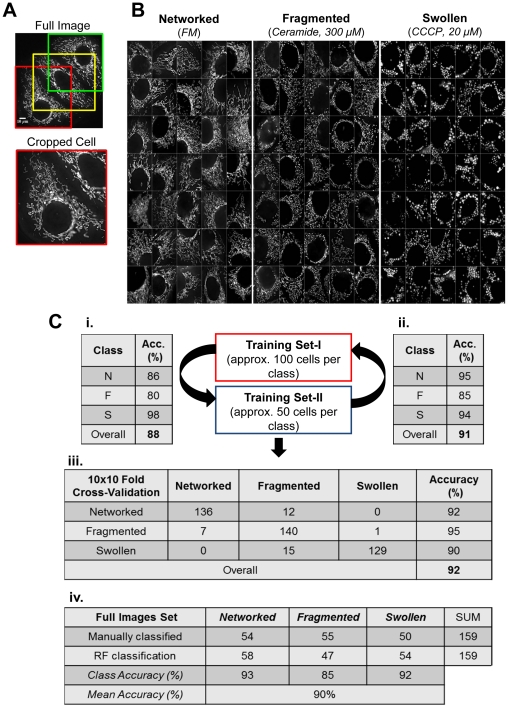
Establishment and validation of the Random Forest (RF) classifier. A) Individual cell analysis- training sets were built from manually-cropped single cells. An example of a “full image” with regions of interest (ROIs) for individual cells and a corresponding “cropped cell” are depicted. B) Phenotypic variability- MCF-7 stably expressing Mito-GFP cells were incubated 6 hours at 37°C with the 3 control conditions: full medium (FM), ceramide (300 µM) and CCCP (20 µM). Shown here are representative examples of mitochondrial phenotypes manually selected for training each class. C) Random Forest classifier- (i) The classification algorithm was first trained with training set I (approx. 100 cropped cells per class) and tested in training set II (approx. 50 cropped cells per class); (ii) Classifier was trained with training set II and validated on training set I. (iii) Both datasets (set I and set II) were combined and used to train the final model. Each tree in the ensemble was calculated using a subset of cells stratified according to class and experimental origin. A 10×10 fold cross validation gives an overall accuracy of 92%. (iv) Comparison of results obtained for individual cells manually classified and the same set of cells automatically classified in their original image by our classifier. For comparison purposes, RF results were reduced to one class showing the major score from Networked/Fragmented/Swollen (N/F/S) intracellular populations.

We developed a supervised learning approach using an image set of cells, which were individually cropped and manually classified as networked, fragmented, or swollen (Figure 2A and B). These image sets were obtained from control conditions (FM, ceramide and CCCP) and submitted to the CellProfiler pipeline (see Information S1 for the detailed description). The extracted features were used to build a Random Forest (RF) classifier. RF method [24] is an established classification algorithm that shows a very robust and competitive performance on diverse data sets. The algorithm is an extension of the bagging principle [25], a method for improving results of machine learning classification, and consists of a collection of classification trees. Two training sets of cropped and manually classified cells were used to build and validate the RF classifier (see Figure 2C). In order to compare our manual classification with the RF classification, we assigned one class, i.e., networked, fragmented, or swollen, per cell, since it is impossible to clearly define intermediate classes within a single cell manually. Therefore, the class with the highest percentage (major score) was considered for validation purposes (Figure 2C). Training sets were crossed-validated and resulted in 92% overall accuracy (Figure 2Ciii).

**Figure 3:**
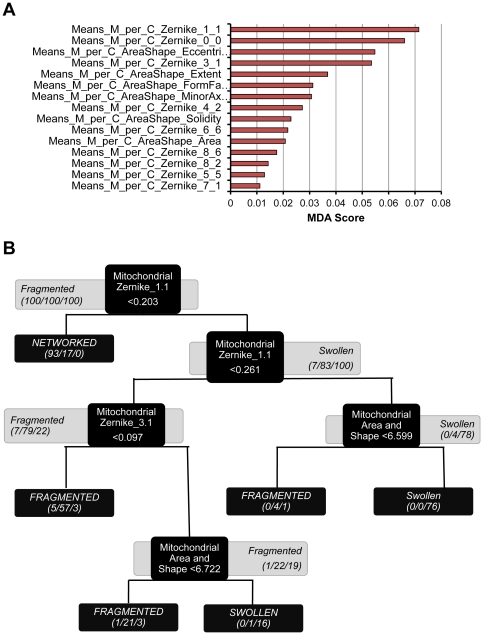
Extracted features and classification. A) MDA score- features used to build the Random Forest (RF) classifier ordered by its mean decrease in accuracy (MDA) value (%). Here we present the most relevant 10 CellProfiler features resulting from the 10×10 fold cross-validation. B) Representative decision tree- Tree consists of fork nodes, each labeled with an attribute and an intermediate class decision, and leaf nodes representing the final morphology classes (N/F/S). Feature/Split selection for the tree building process was weighted by the respective cost (1/MDA) score.

**Figure 4:**
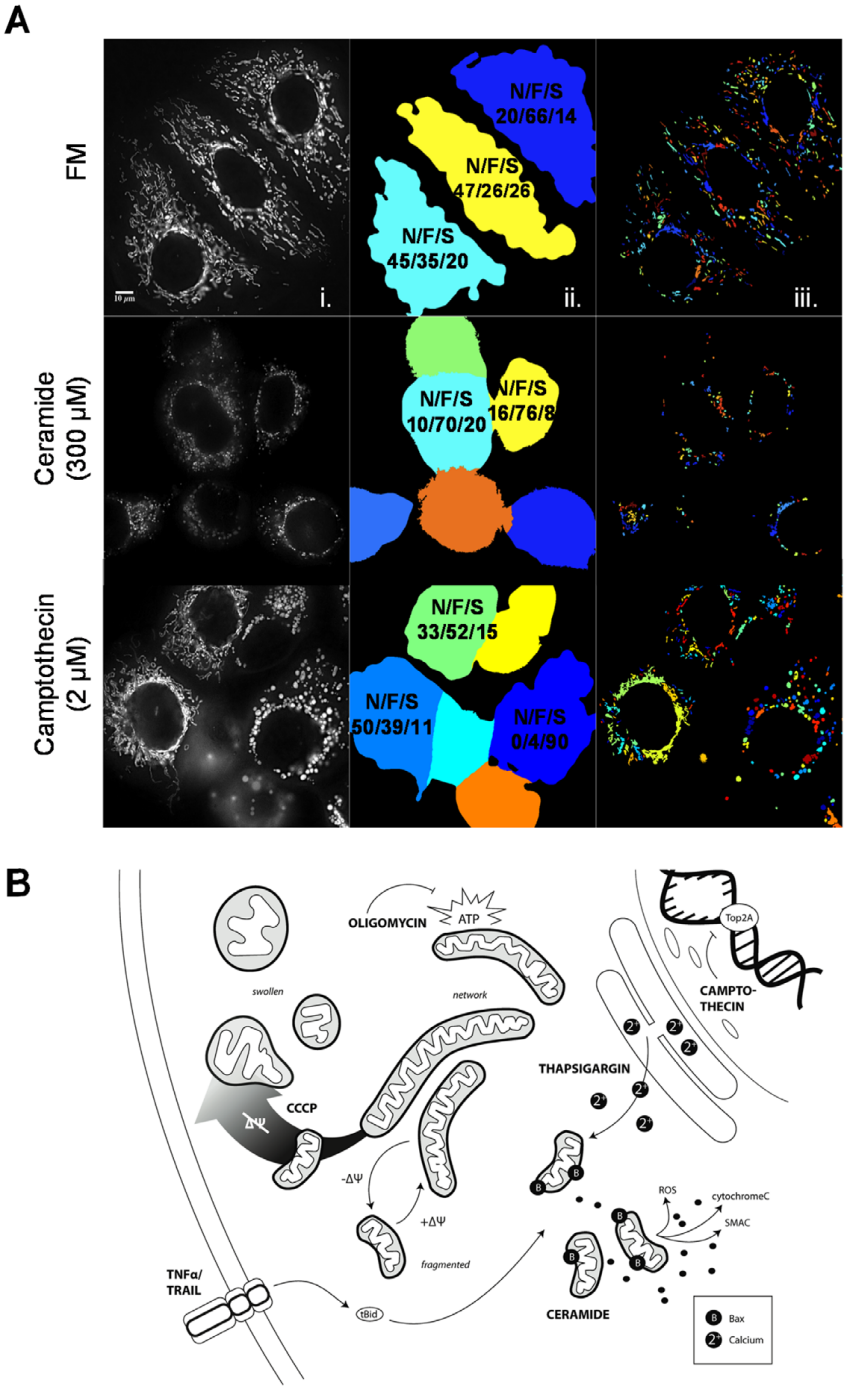
Population wide analysis of mitochondrial morphology. A) CellProfiler pipeline applied to “Full Images”- (i) Representative examples of images as obtained by the DVRT microscope after deconvolution; (ii) Cell border identification and segmentation with final classification for each considered cell (color code corresponds to single cell segmentation); (iii) Mitochondrial segmentation per cell (color code corresponds to single mitochondrion segmentation). Here we present examples for 6 hours incubation (37°C) in control condition (FM) and drug conditions (ceramide, 300 µM, camptothecin, 2 µM). Percentage values of Networked/Fragmented/Swollen (N/F/S) mitochondria are attributed to each cell. These values (N/F/S) can be averaged per cell to obtain whole cell population shifts on mitochondrial morphology under the tested condition. B) Cartoon scheme representing the tested apoptotic drugs and its targets. MCF-7 stably expressing Mito-GFP were incubated for 6 hours at 37°C with 7 different apoptotic drugs inducing a variety of cellular stress: calcium overload (thapsigargin, 1 µM); DNA synthesis inhibition (camptothecin, 2 µM); ATP synthesis inhibition (oligomycin, 10 µM); death receptor (DR) pathway activation (TNFα, 43 ng/mL and TRAIL, 20 ng/mL); mitochondrial fragmentation (ceramide, 300 µM); as well as a mitochondrial uncoupler (CCCP, 20 µM). The scheme summarizes the subcellular impact of our drug selection and depicts the three possible morphologic states of mitochondria: networked, fragmented and swollen. For example, DR activation activates pro-apoptotic tBid, which leads to Bax activation at the mitochondria. Mitochondria are shown in a fragmented state during cytosolic release of pro-apoptotic signaling factors and related to a swollen stated upon loss of ΔΨ_m_ (gradient arrow).

**Figure 5:**
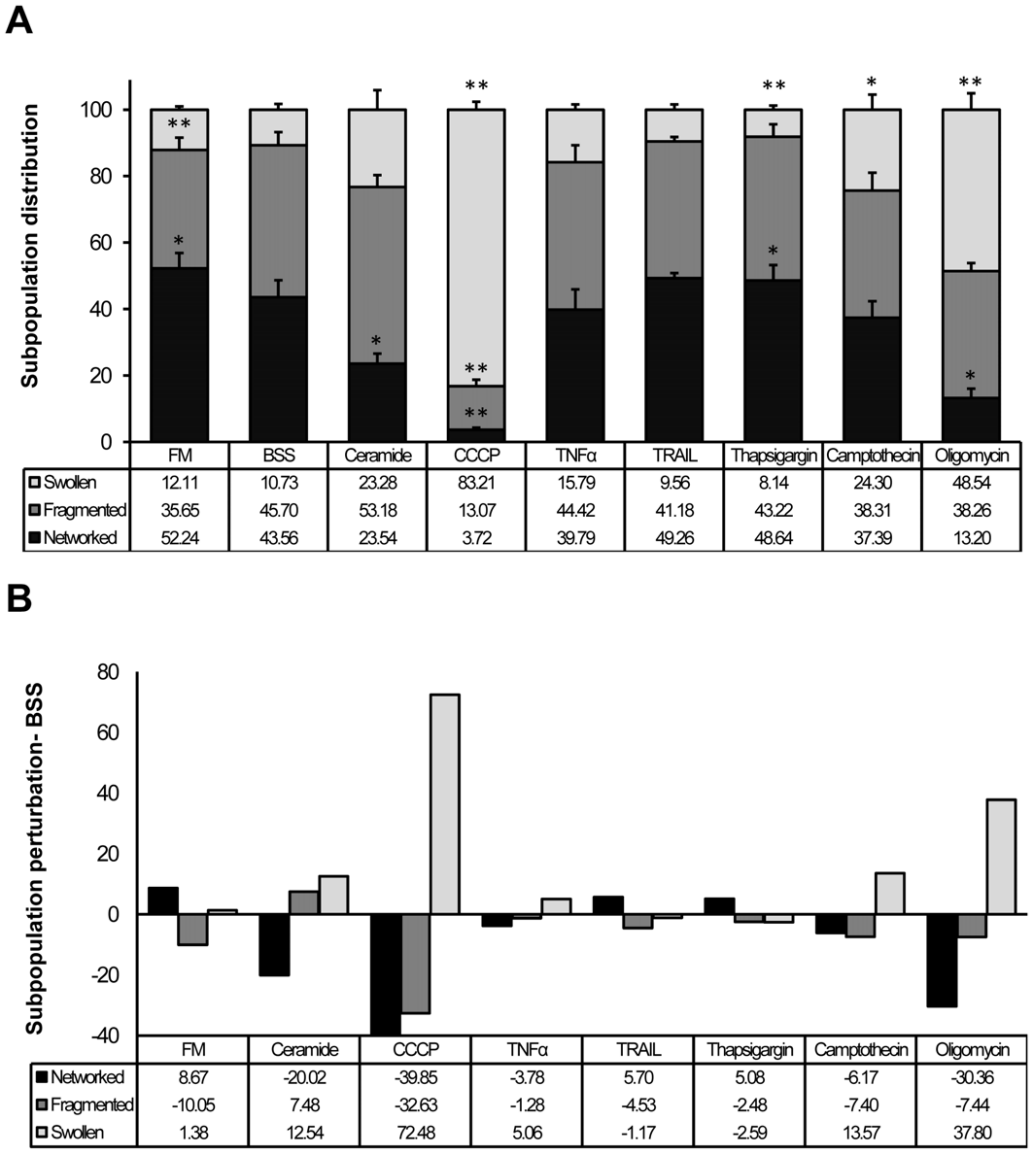
Mitochondrial morphologic classes quantification in response to apoptotic stimuli. A) Mitochondrial classes distribution during apoptosis- Column chart shows the Random Classifier (RF) classification into networked (black), fragmented (gray) and swollen (white) (N/F/S) for the different conditions. Values are given as mean percentage ± s.e.m. of N/F/S per cell for each N. (N = 3, approx. 300 cells per condition; *, P≤0.05, * *, P≤0.01, t-test against BSS). B) Normalization with control- results plotted in A are here normalized against BSS.

While complex ensemble models offer a high accuracy, human interpretation of the model is not feasible. To aid interpretation of the single tree representation of our RF classifier, we utilized the Mean Decrease in Accuracy (MDA) score (Figures 3 and S1) [25]. This score is a measure for feature importance in the RF model. Scalings were applied when considering splits for the representative tree (Figure 3B), and the improvement on splitting on a variable is weighted by its cost (1/MDA) in deciding which split to choose. Our results demonstrate that the Zernike and mitochondrial “Area and Shape” features were the most relevant for our classification (Figure 3). Furthermore, “networked” is the most distinct mitochondrial class, followed by “fragmented” and “swollen” classes, where the mitochondrial “Area and Shape” is determinant for deciding between these two classes (Figure 3B). The representative tree was not used for classification purposes; all classification results used for further analysis were obtained from the RF model built upon the training sets (Figure 2). Thus, the prediction of our classifier was (i) not substantially biased for new cells, which did not undergo manual classification (e.g. Figure 4A and 5), and (ii) describes mitochondrial intracellular heterogeneity by assigning a degree of belonging (RF score) to each class for each cell (%(N/F/S)/cell) (Figure 4A). This is based on the percentage of trees in the ensemble voting for a specific class, and is the basis for all further analysis steps.

### Automated identification of mitochondrial morphology classes within single cells and within populations of cells

The feature extraction pipeline generation was optimized for the morphological classes using cropped single cells, manually classified as networked, fragmented, or swollen phenotypes (Figure 2A and B). Next, this pipeline was applied to full images that had not undergone manual cropping. To determine the classifier accuracy on raw images, a new dataset consisting of randomly chosen images from different conditions was assembled. From this set, 159 individual cells with an obvious phenotype were manually classified, and the manual classification of single cells was assessed against RF classification of the same cells within their original raw images (Figure 2Civ). Once again, for comparison with the manual classification, we considered only the major score (highest % (N/F/S)) present in each cell. Our method presents comparable results when automatically classifying individual cells within full images to those manually classified (90% accuracy; Figure 2Civ).

In summary, the generated pipeline was accurate when applied to images containing multiple cells (Figure 2Civ and 4A) and was able to quantify the mitochondrial morphology response as a function of perturbation-induced shifts of networked, fragmented, and swollen subpopulations (Figure 5). Moreover, we were able to distinguish the several states of mitochondrial morphology within a single cell and provide a quantitative index of intracellular heterogeneity (Figure 4A). In Figure 4A we show representative examples of segmented cells and mitochondria by CellProfiler and respective cell-based RF classifications. The classifier attributes a degree of belonging to each of the three main classes (N/F/S) for each cell and these three values are always taken into consideration, averaged per class over cell population within each condition. Initial conditions revealed a high degree of intracellular (Figure 4A) and population-based heterogeneity (Figure 5 and Table S4), with fragmented and swollen mitochondria co-occurring within a single cell (Figure 4A, segmented yellow cell in FM) and all classes co-occurring within a population (Figure 4A, camptothecin conditions).

### Population analysis of mitochondrial morphology dynamics in response to diverse apoptotic stimuli

We next quantified redistributions of morphology subpopulations in response to various pro-apoptotic stimuli. Cells were treated with compounds known to impact mitochondrial bioenergetics and induce mitochondrial apoptosis (Figure 4B). It is important to note that our experimental model, MCF-7 breast cancer cells, lack caspase 3 [26], and therefore undergo a slower progression of cell death. This allows for an optimal visualization and analysis of mitochondrial morphology in early apoptotic stages, before cells begin to shrink and detach.

Drugs were selected which initiate mitochondrial apoptosis in a spatially heterogeneous manner. Death receptor (DR) ligands TNFα (43 ng/mL) and TRAIL (20 ng/mL) activate the mitochondrial death pathway via caspase 8-mediated cleavage of Bid [27]. The ER calcium pump inhibitor thapsigargin (1 µM) induces ER stress, cytosolic calcium, and subsequent activation of BH3-only proteins [28]. Camptothecin (2 µM), a DNA topoisomerase I inhibitor induces mitochondrial apoptosis [29]. Bioenergetic perturbations were induced with oligomycin (10 µM), which inhibits oxidative phosphorylation at the mitochondrial ATP synthase [30] (Figure 4B).

Images were acquired following 6 hours treatment at 37°C and approximately 300 cells per condition were classified (Figure 5A). Plotted results reflect the drug impact on mitochondrial subpopulations, as (N/F/S) percentages are all taken into account and averaged throughout whole cell population for each experimental N (Figure 5). In parallel to the apoptotic conditions, cells were incubated with two control conditions: FM and BSS. Under FM conditions, mitochondria were mostly networked ((N/F/S)±s.e.m.) = (52.24±4.62/35.65±3.70/12.11±0.98)%). Cells incubated in BSS showed markedly changes ((N/F/S)±s.e.m.) = (43.56±5.11/45.70±4.00/10.73±1.74)%), with increased fragmentation and decreased networked mitochondria (Figure 5). CCCP revealed a 72% increase in the population of swollen mitochondria compared to the control (BSS) (Figure 5B). Curiously, ceramide incubation resulted in a small increase of fragmented mitochondria ((N/F/S)±s.e.m.) = (23.54±3.03/53.18±3.59/23.28±5.86)%) when compared with BSS, although remained the condition with the largest population of fragmented mitochondria (Figure 5). We detected subtle but distinct responses of mitochondrial morphology distribution in comparison to BSS for cells treated with TNFα ((N/F/S)±s.e.m.) = (39.79±6.12/44.42±5.11/15.79±1.61)%) and TRAIL ((N/F/S)±s.e.m.) = (49.26±1.57/41.18±1.33/9.56±1.57)%). While the population of swollen mitochondria increased for TNFα, networked mitochondria remained unchanged in response to TRAIL. Oligomycin considerably increased the number of swollen mitochondria ((N/F/S)±s.e.m.) = (13.20±2.87/38.26±2.39/48.53±4.91)%). Thapsigargin treated cells exhibited a high percentage of networked mitochondria with fragmentation and swollen populations smaller than the control ((N/F/S)±s.e.m.) = (48.64±4.62/43.22±3.74/8.14±1.26)%) (Figure 5B). Camptothecin treatment resulted in similar distributions of networked and fragmented mitochondria ((N/F/S)±s.e.m.) = (37.39±4.96/38.31±5.33/24.30±4.55)%), with a 13% increase of swollen mitochondria when compared to BSS control. Based on intercellular variances (standard deviation, StDev) camptothecin showed the highest intercellular heterogeneity after treatment (StDev = 25%), while CCCP revealed the least population heterogeneity (StDev = 15%) (Table S4).

Mitochondrial dysfunction can drive changes to morphology, and interactions between the mitochondrial morphology machinery and the Bcl-2 family contribute to MOMP. Paradoxically, pro-apoptotic Bax not only activates mitochondrial permeability transition (MPT) when active [31], but can also promote mitochondrial fusion in its inactive form [32]. In order to explore changes to mitochondrial morphology in the context of apoptosis, we determined the impact of the drugs employed above on mitochondrial membrane potential (ΔΨ_m_), Bax activity, cytochrome *c* release and cell death.

### MPT as a measure of cell sensitivity to apoptotic stimuli

Tetramethylrhodamine methyl ester (TMRM), a fluorescent lipophilic cation that electrophoretically accumulates in mitochondria [33], can be photoactivated to generate reactive oxygen species (ROS) levels within the mitochondrial matrix that are sufficient to trigger MPT [34]. Following 6 hours incubation with pro- apoptotic compounds, MCF-7 wt cells were loaded with TMRM (25 nM) for 25 minutes at 37°C. Continuous fluorescence imaging was performed for 5 minutes to induce ROS-dependent triggering of the MPT [35]. The time of ΔΨ_m_ loss reports the threshold for MPT induction, and can be used as a gauge for mitochondrial sensitivity to specific stresses [36].

**Figure 6:**
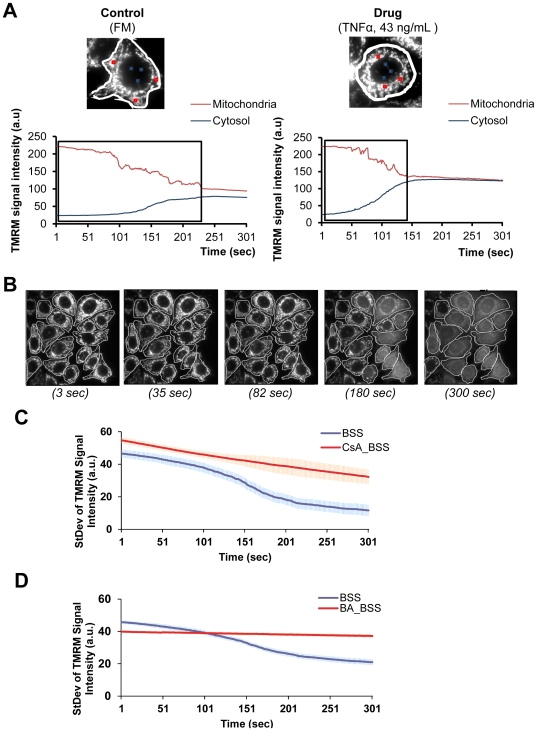
Mitochondrial membrane sensitivity to apoptotic stimuli. A) Mitochondrial membrane potential (ΔΨ_m_)- MCF-7 wild-type (wt) cells were incubated with tetramethyl rhodamine methyl-ester (TMRM, 25 nM) for 25 minutes at 37°C after 6 hours treatment with the apoptotic drugs. Sequential images of TMRM fluorescence were acquired every second using exposure times of 20 milliseconds, during a total of 5 minutes. Here we show the TMRM signal over the 5 minutes time for one cell incubated under full medium (FM) control condition and a cell incubated with a drug (TNFα, 43 ng/mL). The curve plots represent the TMRM signal variation over time in a mitochondrial region (red) and in the cytosol/nucleus region (blue)- here is shown the mean value from the 3 regions depicted in the image. B) Standard deviation (StDev) of TMRM signal- Distribution of the TMRM signal throughout the whole cell was followed over time by the StDev value that corresponds to the standard deviation of the average gray values within the ROI selection (each individual cell, in white). A single focal plane for 3, 35, 82, 180 and 300 seconds in cells under FM are shown. C) Cyclosporin A (CsA)- MCF-7 wt cells were treated with CsA (5 µM, 30 minutes) prior to 6 hours incubation in BSS, followed by TMRM addition as described above. D) Bongkrekic acid (BA)- MCF-7 wt cells were treated with BA (50 µM, 1 hour) prior to 6 hours incubation in BSS, followed by TMRM as described. (Mean values ± s.e.m. per condition are shown; experiments N = 2, approx. 100 cells per condition were followed).

Initially, mitochondria appeared as homogeneously polarized and then entered a phase of stochastic flickering, i.e. transient redistribution of TMRM (Figure 6A). Eventually, ΔΨ_m_ collapsed within mitochondrial populations (Figure 6B and C). In Figure 6A, representative examples are shown for single mitochondria after control or drug treatment (TNFα). By following the TMRM signal intensity along time in mitochondrial areas (mean signal intensity plotted in red) or cytosolic regions (mean signal intensity plotted in blue) a gradual decrease in mitochondrial-TMRM occurs, concomitant with an increase in the cytosolic-TMRM signal. To quantify the kinetics of TMRM release, we measured the StDev [20] of the TMRM signal in individual cells (Figure 6B–D and S5) by considering the whole cell area and extracting a StDev value per cell. Approximately 400 cells per condition were analyzed per condition and the mean value was plotted (Figure 6C, D and S5). In Figure 6C is shown an example of the StDev measurements over time for BSS (see Figure S5 for all conditions).

Cyclosporin A (CsA) and bongkrekic acid (BA) are two commonly used MPTP inhibitors, which act at different sites. CsA binds to the cyclophilin D and BA inhibits at the ANT (ATP/ADP translocator) (for review see [37]). MCF-7 cells were treated with either CsA (5 µM, 30 minutes) or with BA (50 µM, 1 hour) before 6 hours incubation in BSS. Results show that CsA caused a delay in depolarization events and BA blocked mitochondrial depolarization when compared to BSS alone (Figure 6C and D).

**Figure 7:**
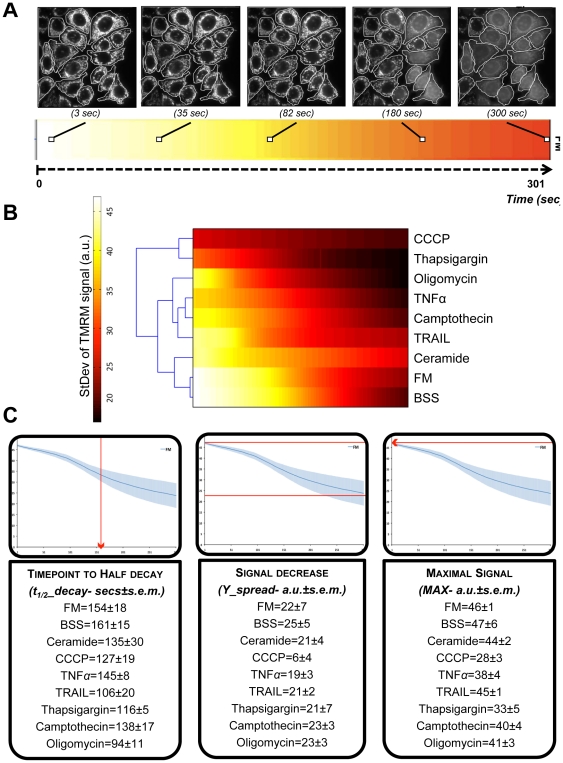
ΔΨ_m_ loss and derived dataset. **A**) Standard deviation (StDev) of TMRM signal as a heatmap- mean values of StDev were translated into a heatmap (color scaled from 0 to 50 in arbitrary units, a.u.). B) Heatmap of StDev values over time for all conditions and derived dendogram (left side diagram in black) illustrates the similarity of responses (Euclidean clustering). C) Derived dataset- Tree parameters were extracted per each StDev curve: 1. t_1/2__decay- time that takes for the signal to reach half of its initial value; 2. Y_spread- Total decrease of the signal over time; 3. MAX- The initial maximum value. These parameters were extracted by using of a MATLAB function (see METHODS for details). Boxes show a representative curve example for the StDev curve of FM over time (301 seconds). (Mean values ± s.e.m. per drug are shown; experiments N = 4, approx. 400 cells per condition were followed).

Signal dissipation curves (Figure S5) were represented as heatmaps (Figure 7A and B) to allow an easy comparison between the drugs. As expected, under FM (negative control), TMRM signal dissipation occurred at the latest point (approx. 232 seconds). Euclidean clustering of our results (Figure 7B) suggests three main groups: 1) conditions which did not impact initial mitochondrial polarization state, 2) drugs which sensitized mitochondria to depolarization, and 3) drugs which depolarized mitochondria.

The latter was apparent for CCCP (20 µM) and thapsigargin (1 µM), in which ΔΨ_m_ was collapsed at the onset of the experiment. In the intermediate group, TRAIL (20 ng/mL), camptothecin (2 µM), oligomycin (10 µM) and TNFα (43 ng/mL) similarly sensitized mitochondria to depolarization events. As expected, under control FM and BSS conditions, ΔΨ_m_ loss occurred at later time points than for most drug treatments. Surprisingly, ceramide (300 µM) clustered together with control conditions. Although the initial StDev of the TMRM signal was low, ceramide revealed a very mild impact on mitochondrial membrane depolarization over time. For further analysis, the dynamic response was described by extracting three subset features: the half time for the signal-StDev decay (t_1/2__decay); the spread of the signal-StDev (Y_spread) and the maximum initial signal-StDev value (MAX) (Figure 7C).

### Apoptotic compounds result in different levels of Bax activation

**Figure 8:**
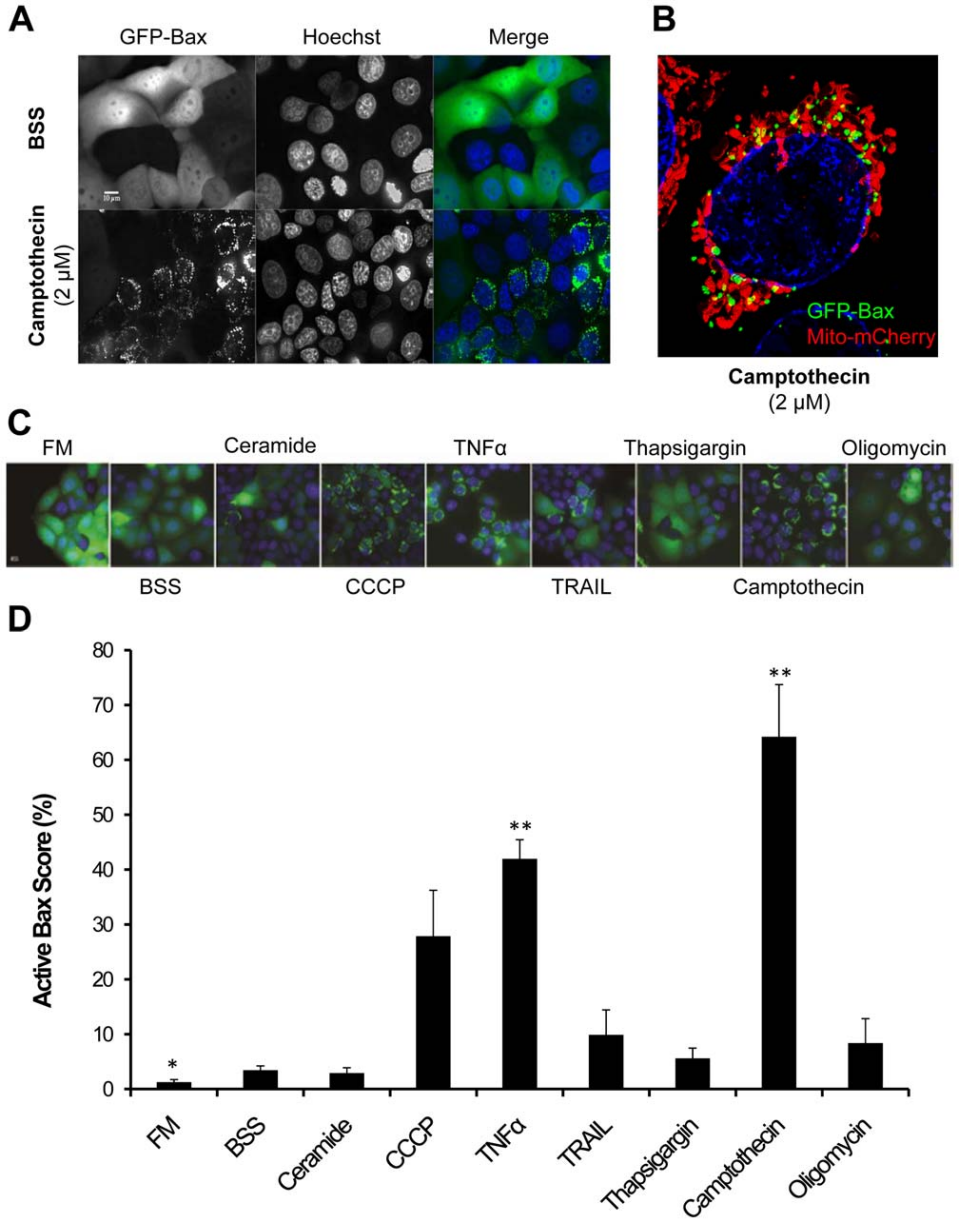
Bax clustering under apoptotic stress. A) Bax clustering- MCF-7 cells stably expressing GFP-Bax were incubated 6 hours at 37°C with the different conditions and nuclei were stained with Hoechst (100 ng/mL). Here is shown a representative example of basal levels of Bax activation (BSS) and an example of Bax activation under camptothecin (2 µM). B) Active Bax translocates to the mitochondria- MCF-7 cells stably expressing GFP-Bax were transiently transfected with mito-mCherry and incubated 6 hours (37°C) with camptothecin (2 µM) (Hoechst for nuclei). The 3D rendering (ImageJ) image shows GFP-Bax (green) translocated to mitochondria (in red). C) Bax clustering- Representative microscope region for each pro-apoptotic condition is shown. D) Bax levels- Cells with GFP-Bax clusters were scored as “positive” for Bax activation. D) Cells “positive” for activated Bax were scored and plotted as shown. Values are presented as mean percentage ± s.e.m. (N = 5, approx. 500 cells/condition; *, P≤0.05, * *, P≤0.01, t-test). Images were acquired with a DVRT scope and a 40× Objective.

Bax has been shown to both promote mitochondrial fusion [32] and participate in fragmentation events [38]. Drp1, which promotes fission, can enhance Bax activation and cytochrome *c* release [39]. On the other hand, pro-fusion protein, Mfn2, was shown to block Bax activity [8]. We therefore measured Bax activity in response to drug treatments. MCF-7 cells were stably transfected with GFP-Bax, which upon activation forms high molecular weight clusters (Figure 8) [40]. Under BSS control conditions (Figure 8A, SBB), GFP-Bax was homogeneously distributed within the cytosol, with low basal activation (5% shown in Figure 8D). In response to apoptotic stimuli, GFP-Bax became punctate and clustered at the mitochondria (Figure 8A and B). Following 6 hours of treatment with CCCP (20 µM), TNFα (43 ng/mL), or camptothecin (2 µM), 30% to 80% of the cells showed GFP-Bax clustering (Figure 8D). Low levels of GFP-Bax clustering were observed in response to ceramide (300 µM), thapsigargin (1 µM) and oligomycin (10 µM) (Figure 8D). Notably, the two DR ligands, TNFα (43 ng/mL) and TRAIL (20 ng/mL), showed marked differences in Bax activation (Figure 8). While both TNFα and TRAIL treatments resulted in a relatively small population of fragmented mitochondria (Figure 5), TNFα increased the number of swollen mitochondria, and TRAIL maintained a high population of networked mitochondria (Figure 5B). Moreover, although both treatments increased Bax activation, the response was about 4-fold higher with TNFα than with TRAIL (Figure 8C and D). We have assessed cytochrome *c* release under control and drug conditions (Figure S3), and cells expressing active GFP-Bax (clusters) exhibited loss of mitochondrial cytochrome *c* (Figure S3). Finally, we quantified cell death at 6 hours of treatments, and observed that cell death was minimal under most conditions (Figure S4). At this timepoint, only camptothecin caused significant cell death, in accordance with its induction of high levels of Bax activation (Figure 8D and S4). For the majority of the conditions, cell death was only evident at the later time point of 24 hours (Figure S4).

### Fuzzy Logic (FL) analysis of mitochondrial morphology and cell death datasets

**Figure 9:**
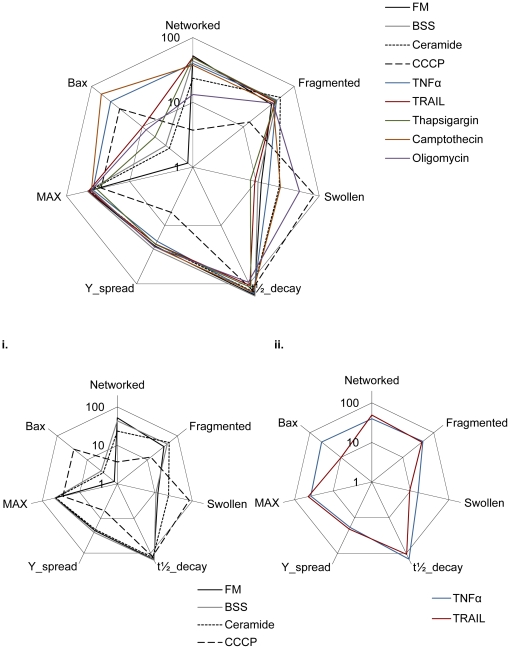
Ensemble of parameters extract from imaging datasets. Data concerning mitochondrial morphology classification, Bax activation scores and ΔΨ_m_ derived dataset were acquired for matched apoptotic condition after 6 hour incubation at 37°C. Shown are the ensemble datasets plotted together as a radar plot. This approach illustrates how each of the acquired parameters (mitochondrial morphology classes, ΔΨ_m_ subset and Bax activation) varies among tested conditions. i) Subset of results for control morphology conditions: FM, BSS, ceramide and CCCP. ii) Subset of results for death receptor ligands, illustrating. It is possible to observe distinct mitochondrial responses of TNFα and TRAIL.

In summary, our results above show no apparent linear relationship between morphology, ΔΨ_m_, or Bax activation (Figure 9), suggesting that more complex interactions exist between mitochondrial morphology and apoptotic events. Therefore, we performed computational modeling to suggest cause-and-effect relationships between morphological and functional features of mitochondria in response to cell death activation. Primary and secondary metrics contain biologically relevant information, yet are not possible to incorporate using mechanistic modeling frameworks such as ordinary differential equations (ODE) due to lack of knowledge of the underlying interactions at the molecular level. Fuzzy logic (FL) is a rule-based approximate artificial reasoning method suitable for investigating signal transduction pathways [41]. FL-based approaches allow for the integration of prior knowledge and experimental data enabling high interpretability [42]. Here, FL was used for the analysis of the multivariate, heterogeneous datasets described above.

Initially, we performed an exhaustive search for all possible interactions by constructing 30 single input-single output (SISO) FL models. Each interaction represents a potential cause-and-consequence relationship. In order to assemble a SISO FL model, we used two membership functions (MFs) to represent the single input in our fuzzy system, and combined them linearly upon aggregation of two rules per model. This stepwise linear combination allowed for the simulation of nonlinearity. A parameter distribution mimicking the structure of a neural network (NN) enabled the use of learning algorithms [21]. Importantly, this eliminated the bias inherent to manually implementing the model. The SISO model was then fit to the data. An advantage of this method is that it is straightforward to extend the approach to a multiple input-single output model (see Figure S2 and Information S1 step 2 for detailed description).

**Figure 10:**
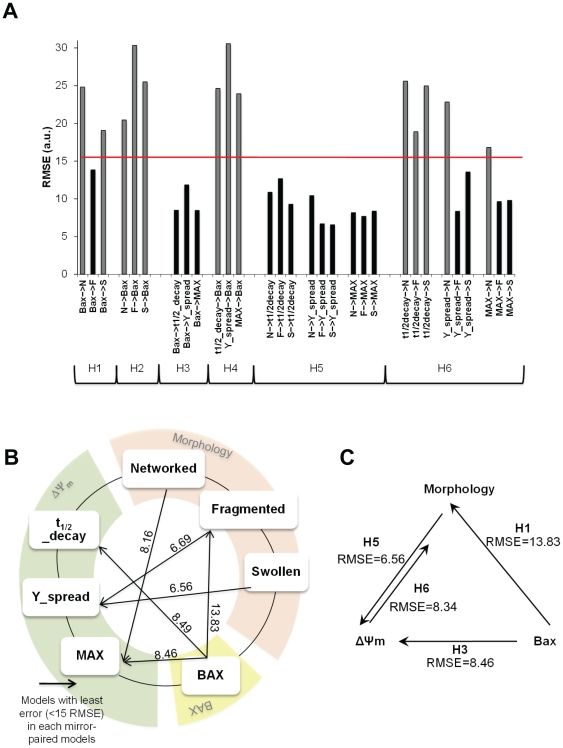
Fuzzy Logic modeling. A) Root mean square error (RMSE) of all 30 models. After assembly, all Single Input-Single Output (SISO) models were trained with the data for the corresponding interaction. Model accuracy was measured upon calculation of RMSE. Here are plotted the RMSE for all possible hypothesis (H): H1. “Individual mitochondrial morphology classes cause Bax”; H2. “Bax is responsible for morphology classes”; H3. “Bax causes each of mitochondrial membrane potential (ΔΨ_m_) subset”; H4. “ΔΨ_m_ subset induces Bax”; H5. “Mitochondrial morphology induces ΔΨm subset”; H6. “Each of the ΔΨ_m_ subset is responsible for the morphologic classes”. First selection was made by discarding all models with a RMSE>15 (threshold in red). Secondly, the least errors between “mirror-models” were chosen (black bars). For clarity, H1-model is the “mirror-model” of the H2-model as H3-model is the opposite of H4-model and as H5-model is for H6-model. B) Detailed causality predictions between datasets- Scheme representing the final 6 most relevant predictions out of the 30 models. To assign these directional arrows, associated RMSE errors of the individual “mirror-models” were compared, e.g. H1-RMSE against H2-RMSE. Arrow direction was chosen based on the smaller error between “mirror-models” per dataset: Morphology, Bax and ΔΨ_m_. The numeric values associated with the arrows correspond to the actual RMSE value resultant for the directional model prediction. C) Simplified scheme summarizing main interactions and causality suggested by our modeling results.

To determine directionality of all interactions (relationships between morphological and functional responses), we analyzed each model in a pair-wise manner (Figure 10A): the two analogous models encoding the two potential senses are termed “mirror-models”, e.g. the models which considered Bax influence on mitochondrial morphology classes were compared against the models in which mitochondrial morphology classes influenced Bax activity. From each pair of “mirror-models”, the one with an error (RMSE) higher than 15 were excluded (threshold in Figure 10A). Thereby we obtained a set of models with a defined directionality of input-output (Figure 10C). From the remaining models those with the least error within each “mirror-model” were selected and its direction represented in Figure 10B and C (black arrows for the smaller RMSE). Our exhaustive search results suggest that Bax activation was strongly related to both ΔΨ_m_ and mitochondrial fragmentation, which in turn, strongly influenced ΔΨ_m_ dynamics together with the swollen mitochondrial morphologic state (Figure 10B). In summary (Figure 10C), Bax is suggested to be upstream of mitochondrial depolarization and mitochondrial fragmentation. In turn, mitochondrial morphology and ΔΨ_m_ are closely related in both directions, although with different intensities revealed by a smaller error on the direction from mitochondrial fragmented states (fragmented and swollen) to ΔΨ_m_ (RMSE of approx. 6.60 a.u., discontinuous arrow).

## Discussion

High-resolution imaging is uniquely suited for addressing complex events within single cells. As signaling events do not function in a synchronized binary manner, it was necessary to measure changes at the population level. Here we employed three high-content imaging approaches to access information contained not only at the subcellular level, but also at the population level.

### Bioenergetic and apoptotic events result in diverse mitochondrial morphology

The heterogeneous response of mitochondria to stress allowed for identification of three distinct mitochondrial morphologies and classification of phenotypic responses based on redistributions of subpopulations (Figure 1B). It was critical to include the “swollen” phenotype [43] in our analysis, as it greatly enhanced functional information content, serving as an indicator for bioenergetic dysfunction. Bioenergetic collapse was induced with CCCP, which is presumed to induce mitochondrial swelling by influx of water due to osmotic disruption. Similarly, inhibition of F1F0-ATPase with oligomycin enhanced the swollen subpopulation (Figure 5B). Indeed, swollen subpopulations revealed the greater variation in response to our drug selection, indicating that in a classical classification of merely two phenotypes (networked and fragmented) these swollen mitochondria would be misclassified as fragmented. It is notable that both thapsigargin and CCCP induced a potent physiological impact (Figure 7B), which was not equally reflected by their resultant morphological changes where networked and fragmented mitochondria still coexisted in thapsigargin treated cells (CCCP: ((N/F/S)±s.e.m.) = (3.72±0.58/13.07±1.95/83.19±2.41)%); thapsigargin: ((N/F/S)±s.e.m.) = (48.64±4.62/43.22±3.74/8.14±126)%). Surprisingly, we found no linear correlation between impact on ΔΨ_m_ and mitochondrial morphology. Thapsigargin, which induces an increase in cytosolic calcium [44] and caused ΔΨ_m_ collapse (Figure 7B), would have been expected to enhance mitochondrial swelling [45]. Similarly, oligomycin caused ΔΨ_m_ collapse (Figure 7B) and resulted in an elevated swollen subpopulation (Figure 5B), but with coexistence of fragmented and networked mitochondria ((N/F/S±s.e.m.) = (13.20±2.87/38.26±2.39/48.53±4.91)%). Conversely, ceramide, which had the least impact on mitochondrial membrane depolarization induced an increase in fragmented and swollen subpopulations ((N/F/S±s.e.m.) = (23.54±3.03/53.18±3.59/23.28±5.86)%) when compared with FM condition (Figure 5 and Figure 7B). Interestingly, cells treated with ceramide showed highly fragmented mitochondria when analyzed on a single-cell basis [22], but population-based classification revealed co-existence of swollen and networked population (approx. 22% each). Furthermore, activation of different DRs by TNFα and TRAIL showed subtle but distinct effects (Figure 9ii). Whereas TNFα increased the incidence of swelling, TRAIL-treated cells maintained more networked mitochondria (Figure 5B). This lack of correlation may be due to the dual roles of swelling, both in cytochrome *c* release [46] and cytoprotection [47]. Overall, the different apoptosis inducers differentially impacted morphologies (Figure 5 and Table S4). Surprisingly, fragmentation was not the most prominent phenotype, even under conditions where Bax was considerably high (e.g. TNFα, camptothecin, CCCP). Apoptotic drugs had the strongest impact on the swollen phenotype, suggesting its association with apoptosis (Bax activation) rather than the fragmented state.

### Bax activation did not correlate with fragmentation

To directly address the apoptotic mitochondrial state we measured Bax activation, an apoptotic point-of-no-return that occurs as a single event in the population of mitochondria [19]. GFP-Bax reports a binary cellular response, allowing for precise manual classification of the population response to different apoptotic stimuli. It should be noted that GFP-Bax overexpression in stable cell lines likely sensitized cells to apoptotic stimuli, so that endogenous Bax activity at the 6 hour time point is likely not matched. However, such an approach offers insight into the rate at which Bax is impacted. Three of the drugs tested induced significant Bax activation: CCCP, TNFα, and camptothecin (Figure 8D). Likewise, these were the conditions that induced cell death at 6 hours treatment (Figure S4). Notably there was no apparent relationship between distribution of mitochondrial morphologies and levels of Bax activation (Figure 9). CCCP activation of Bax (28%), suggests that Bax activation is downstream of compromised mitochondrial bioenergetics. Hence, under certain conditions, regulation of mitochondrial morphology can be uncoupled from Bcl-2 signaling. Interestingly, it has been shown that CCCP alone is not sufficient to trigger Bax in MCF-7 wt cells [48], suggesting that our overexpressed Bax-MCF-7 cell line is more sensitive to bioenergetic stress. Nonetheless, oligomycin, which similarly enhanced swollen and reduced fragmented and networked mitochondrial subpopulations had little impact on GFP-Bax (8%). Furthermore, the TNFα and TRAIL receptor ligands, which had a distinctive impact on subpopulation distributions, also acted differently on GFP-Bax activation (TNFα, 42% and TRAIL, 10%) (Figure 8D and 9). These results suggest that TNFα apoptotic signaling to the mitochondrion is faster than via TRAIL signaling.

### Induced- ΔΨ_m_ collapse revealed heterogeneous drug action

The use of dyes is more challenging compared to GFP-based sensors, due to photo-toxicity and loading concerns [49]. We exploited the photo-toxicity effect and used it to locally induce reactive oxygen species (ROS) within the mitochondrial matrix. Because of the heterogeneous sensitivity of mitochondria to MPT activation, we measured multiple parameters of mitochondrial energetic response to stress. As such, single cell (and subcellular) MPT events were quantified and averaged over the population. High cytosolic calcium accumulation induces mitochondrial uncoupling and opening of the MPTP to trigger matrix swelling [50]. Therefore it is not surprising that CCCP (mitochondrial uncoupler) and thapsigargin (responsible for calcium overload) showed very low ΔΨ_m_ at the onset (after 6 hours treatment) and therefore clustered together (Figure 7B). These two drugs differentially impacted both mitochondrial morphology and Bax activation (Figure 9), suggesting that MOMP can occur independent of or primary to Bax activation and mitochondrial swelling as earlier reported [51], [52]. In accordance with MPT association with swollen states, camptothecin, TNFα and oligomycin, three of the drugs presenting mostly swollen mitochondria, triggered MPT to a similarly high extent (Figure 7B). Given that the ΔΨ_m_ drives mitochondrial fusion [53], [54], we expected a negative correlation between initial ΔΨ_m_ (MAX) and time to depolarization (t_1/2__decay) with mitochondrial networked state. Nevertheless, this was true only for control conditions (FM and BSS). For instance, ceramide had little impact on ΔΨ_m_ (Figure 7B and S5) although it reduced networked mitochondria (Figure 5). Similarly, we also expected negative correlations between Bax activation and networked state, yet camptothecin induced both high Bax activation (64%) and showed elevated networked mitochondria (N±s.e.m. = (37.39±4.96)%) (Figure 9).

### Rule-based modeling of collective dataset suggests a hierarchy for mitochondrial apoptotic events

The primary analysis of our compendium of data showed no linear relationship between the different datasets (Figure 9). FL modeling was used to investigate non-linear relationships within datasets through an exhaustive search approach, and learning algorithms were used to fit the model to our data. The resulting models suggest that upon Bax activation, mitochondria become fragmented and that different states of mitochondrial morphology closely relate to MPT (Figure 10B). First, Bax is actively involved in causing mitochondrial fragmentation, consistent with reports that its interaction with mitochondrial fission protein Drp1 regulates fragmented states (Table 1). Secondly, our models suggest that mitochondrial morphology states are tightly linked to MPT dynamics (Figure 10B and C). As previously reported, our model proposes a strong connection between ΔΨ_m_ and non-networked states of mitochondrial morphology, fragmentation in particular ([55], Figure 10C). Indeed, previous studies have shown that by inhibiting mitochondrial fragmentation a delay in MPT is observed (Table 1). Finally, our model correctly predicted that Bax activation is upstream of MPT, consistent with the previously experimentally demonstrated hierarchy [56]. Here the authors demonstrated that Drp1-mediated mitochondrial fragmentation can be downstream of Bax activation, but occurs prior to ΔΨ_m_ loss in Hela cells (Table 1).

**Table 1:** Summary of model predictions.

Causality	Least error parameter (RMSE)	Reported interactions
Bax→Mitochondrial Morphology	*Fragmented* (13.83)	Active Bax redistributes to the mitochondria and stimulates Drp1-mediated fission during apoptosis, leading to fragmentation [[6], [7], [38], [58], [59], [60], [61].
Bax→ΔΨ_m_	*Max* (8.46); *t1/2_decay* (8.49)	Bax undergoes conformational changes and oligomerization resulting in loss of ΔΨ_m_ and subsequent MOMP [19], [31], [56], [62], [63], [64], [65], [66], [67].
Mitochondrial Morphology→ΔΨ_m_ (Y_spread)	*Fragmented* (6.69); *Swollen* (6.56)	Inhibition of fragmentation has been shown to delay MOMP [7], [60], [68]. Disruption of the mitochondrial outer membrane and consequent loss of ΔΨ_m_ can result from intensive swelling [69], [70], [71], [72].

RMSE- Root mean square error; **ΔΨ_m_** - mitochondrial membrane potential; MOMP- Mitochondrial outer membrane permeability.

Here are stated the final most relevant modeling predictions and the literature references that validate them.

Overall, our results demonstrate that the integrated response of the mitochondrion to diverse stimuli is rarely, if ever, linear. Cell-to-cell heterogeneity represents a rich source of biological information, but remains relatively unexploited due to challenges in its detection and quantification. To that end we utilized high-content biosensors and rich feature extraction to quantify subcellular mitochondrial phenotypes, identify single cell dynamics and phenotype distributions in subpopulations of cells. Importantly, fuzzy logic-derived predictions based on these measurements are in accordance with published data, thereby supporting the suitability of our approach for determining the importance and role of mitochondrial network maintenance in the regulation of apoptotic cell death.

Earlier studies have shown that the combination of theoretical and computational approaches with live-cell imaging and quantitative biochemical analysis can provide new insight into apoptotic mechanisms (for recent review see [57]). Our platform, established and validated for human MCF-7 cells, can be extended and readily applied for further mitochondrial-related studies. Namely, by collecting new training and validation sets, mitochondrial morphology in different cell lines can be investigated, as well as new phenotypic classes can be added (e.g. hyperfused [13]). Note that our rule-based model can be readily used to include datasets related to mitochondria function (e.g. respiration levels, degradation by mitophagy) and to cell death events (e.g. calcium overload, DNA fragmentation). Thus, the here-described platform provides a flexible tool to integrate heterogeneous data into a unified analysis and classification pipeline.

## Supporting Information

Figure S1 **Feature weighted importance-** The extracted features are ordered in a descending manner according to their mean decrease in accuracy (MDA) score obtained during the Random Forest (RF) model construction. The RF algorithm estimates the importance of a feature by calculating how much the prediction error increases when the data for that variable is permuted. The calculations are performed tree by tree as the RF is constructed to obtain the final descending order of importance.
(TIF)

Figure S2 **Representative Single input-single output (SISO) model-** Example of one model built upon the hypothesis that Bax activation caused fragmented mitochondria. The parameters of the model are distributed following a neural network (NN) structure. In the first layer are shown the parameters of the membership functions (MFs) that fuzzified Bax activation, mapping the degree of membership (DOM) of its measurements into 2 fuzzy sets. These fuzzy sets represent “low” and “high” levels of Bax activation. The second layer has scalability purposes: it would contain the rules to combine all the inputs if the model had more than 1 input. The third layer contains parameters (c) to linearly combine the ***i*** input MFs. Input and output MF parameters were fitted to the data. The forth layer aggregates the values from layer 3 to finally model the behavior of “fragmented” mitochondria as a function of “Bax”.
(TIF)

Figure S3 **Drug-induced cytochrome** ***c*** **release.** Representative MCF-7 cells stably expressing GFP-Bax and immunostained for cytochrome *c* and COXIV (mitochondria) following 6 hours subjection to control (FM, BSS) or drug conditions. Nuclei were detected using Hoechst (100 ng/mL). Images were acquired with a DVRT microscope and a 63× objective (approx. 60 cells per condition were imaged).
(TIF)

Figure S4 **Cell death dataset.** Cells were plated in 96 well plates, and cell death was quantified for each condition at 6 hours and 24 hours incubation with indicated drugs at 37°C. Dead cells were stained with propidium iodide (PI, 1.0 ug/ml) and signal intensity measured by plate reader (excitation: 530 nm; emission: 620 nm). Results are normalized to control and represented as percentage ± s.e.m (BSS, 100%). (N = 4).
(TIF)

Figure S5 **Quantification of ΔΨ_m_ sensitivity in response to apoptotic stimuli.** MCF-7 wild-type (wt) cells were incubated with tetramethyl rhodamine methyl-ester (TMRM, 25 nM) for 25 minutes at 37°C after 6 hour treatment with the apoptotic drugs. Sequential images of TMRM fluorescence were then acquired every second using exposure times of 20 milliseconds, during a total of 5 minutes. TMRM signal over time is reported as the StDev value, which corresponds to the standard deviation of the average gray values within each individual cell. A) Depolarization profiles of initial conditions. TMRM signal StDev over 5 minutes (301 seconds) for the initial conditions used to build mitochondrial morphology training sets: FM, ceramide (300 µM) and CCCP (20 µM). B) Depolarization profiles of drug selection.- TMRM signal StDev over 5 minutes (301 seconds) for apoptotic conditions: BSS, TNFα (43 ng/mL), TRAIL (20 ng/mL), thapsigargin (1 µM), camptothecin (2 µM) and oligomycin (10 µM). Values are presented as mean ± s.e.m. (N = 4, approx. 400 cells/condition).
(TIF)

Table S1 **List of Features extracted per cell and related to the nucleus.**
(DOCX)

Table S2 **List of Features extracted per cell and related to the cell.**
(DOCX)

Table S3 **List of Features extracted per cell and related to each mitochondrion.**
(DOCX)

Table S4 **Intercellular variances of mitochondrial class subpopulations under the different drug treatments.**
(DOCX)

Information S1 (DOC)
