# Supplementary figures and images for: Multi-Parametric Analysis and Modeling of Relationships between Mitochondrial Morphology and Apoptosis
Source: PLoS One. 2012 Jan 17;7(1):e28694. doi: 10.1371/journal.pone.0028694 (PMC3260148; doi:10.1371/journal.pone.0028694)

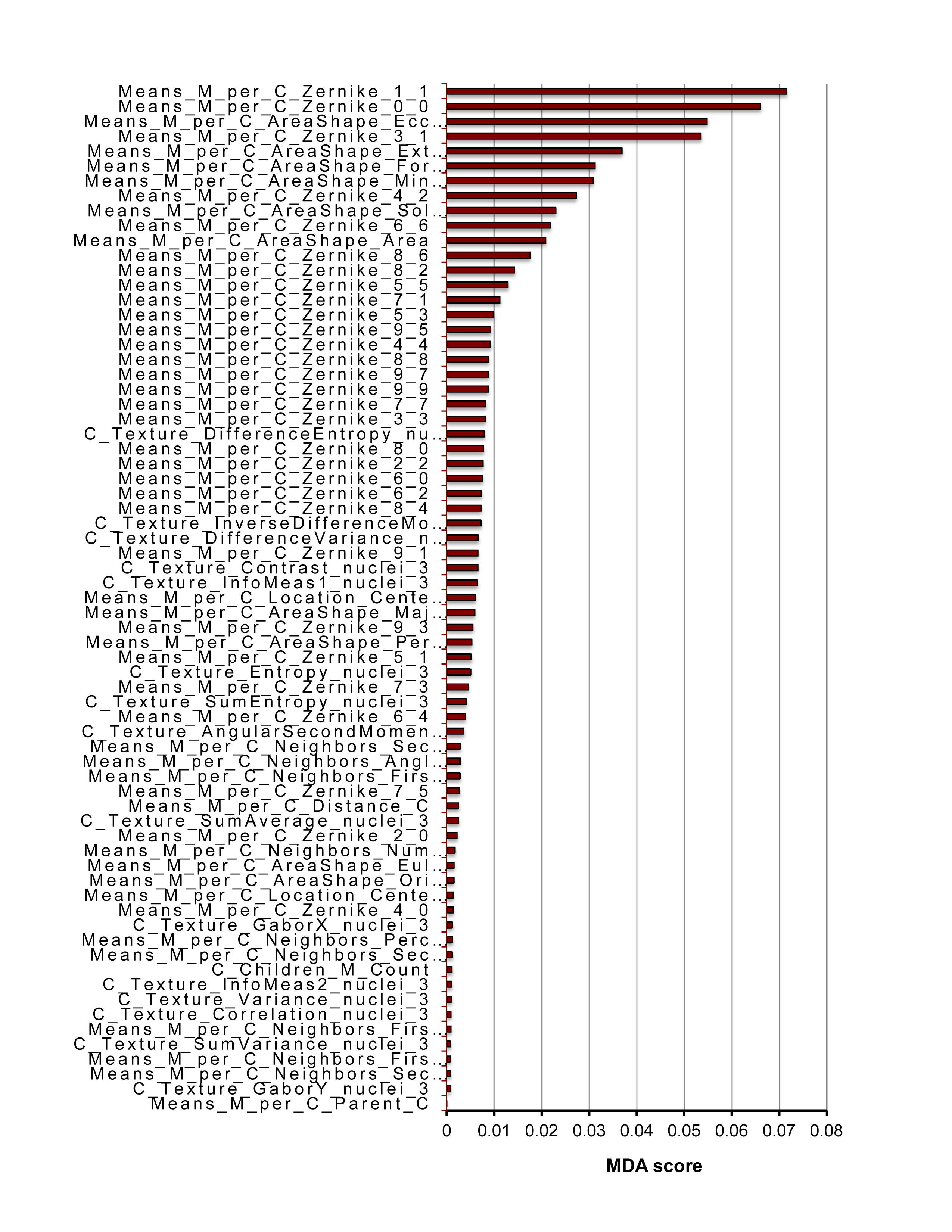

Supplement: Figure S1 — Feature weighted importance- The extracted features are ordered in a descending manner according to their mean decrease in accuracy (MDA) score obtained during the Random Forest (RF) model construction. The RF algorithm estimates the importance of a feature by calculating how much the prediction error increases when the data for that variable is permuted. The calculations are performed tree by tree as the RF is constructed to obtain the final descending order of importance. (TIF) [file pone.0028694.s001.tif]

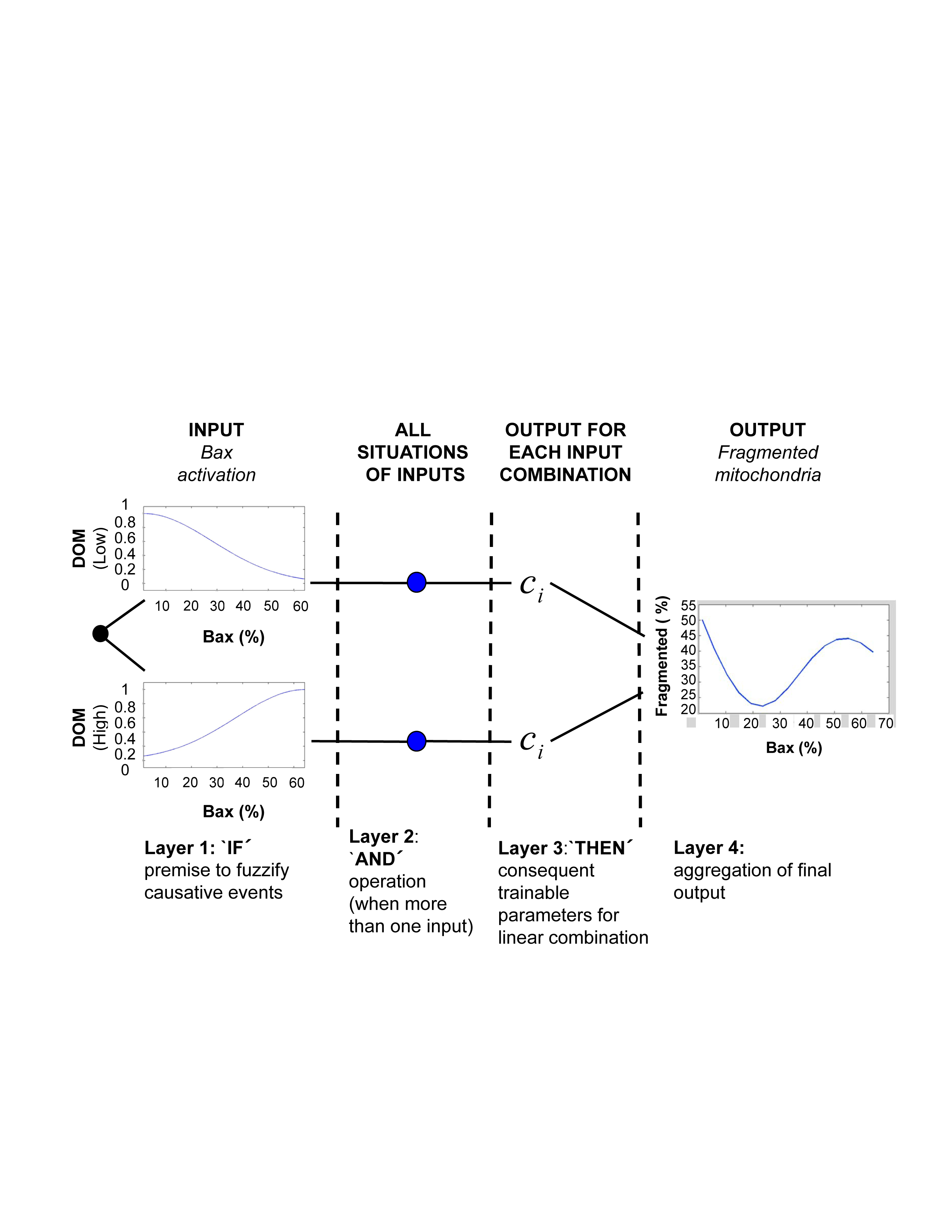

Supplement: Figure S2 — Representative Single input-single output (SISO) model- Example of one model built upon the hypothesis that Bax activation caused fragmented mitochondria. The parameters of the model are distributed following a neural network (NN) structure. In the first layer are shown the parameters of the membership functions (MFs) that fuzzified Bax activation, mapping the degree of membership (DOM) of its measurements into 2 fuzzy sets. These fuzzy sets represent “low” and “high” levels of Bax activation. The second layer has scalability purposes: it would contain the rules to combine all the inputs if the model had more than 1 input. The third layer contains parameters (c) to linearly combine the i input MFs. Input and output MF parameters were fitted to the data. The forth layer aggregates the values from layer 3 to finally model the behavior of “fragmented” mitochondria as a function of “Bax”. (TIF) [file pone.0028694.s002.tif]

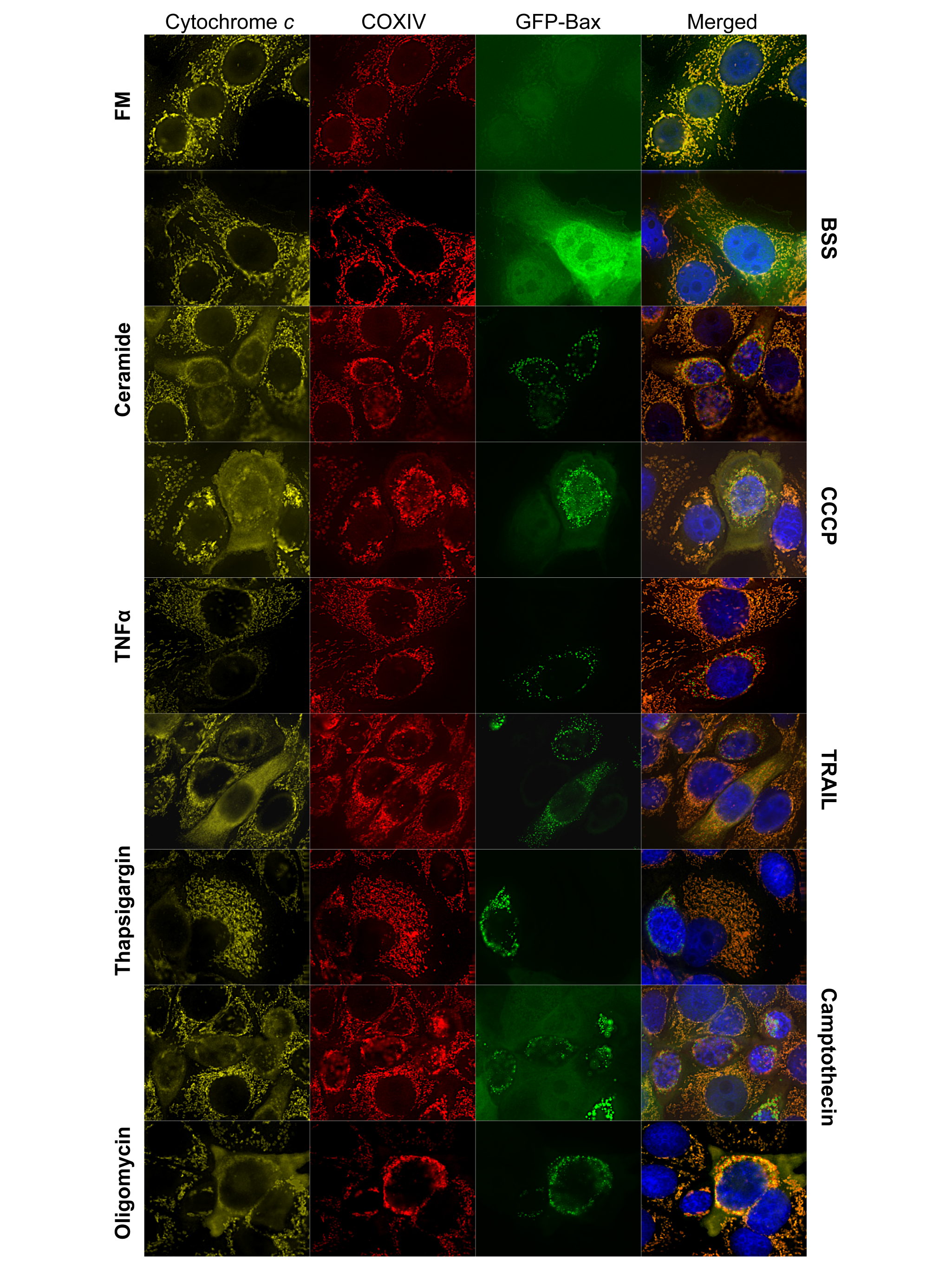

Supplement: Figure S3 — Drug-induced cytochrome c release. Representative MCF-7 cells stably expressing GFP-Bax and immunostained for cytochrome c and COXIV (mitochondria) following 6 hours subjection to control (FM, BSS) or drug conditions. Nuclei were detected using Hoechst (100 ng/mL). Images were acquired with a DVRT microscope and a 63× objective (approx. 60 cells per condition were imaged). (TIF) [file pone.0028694.s003.tif]

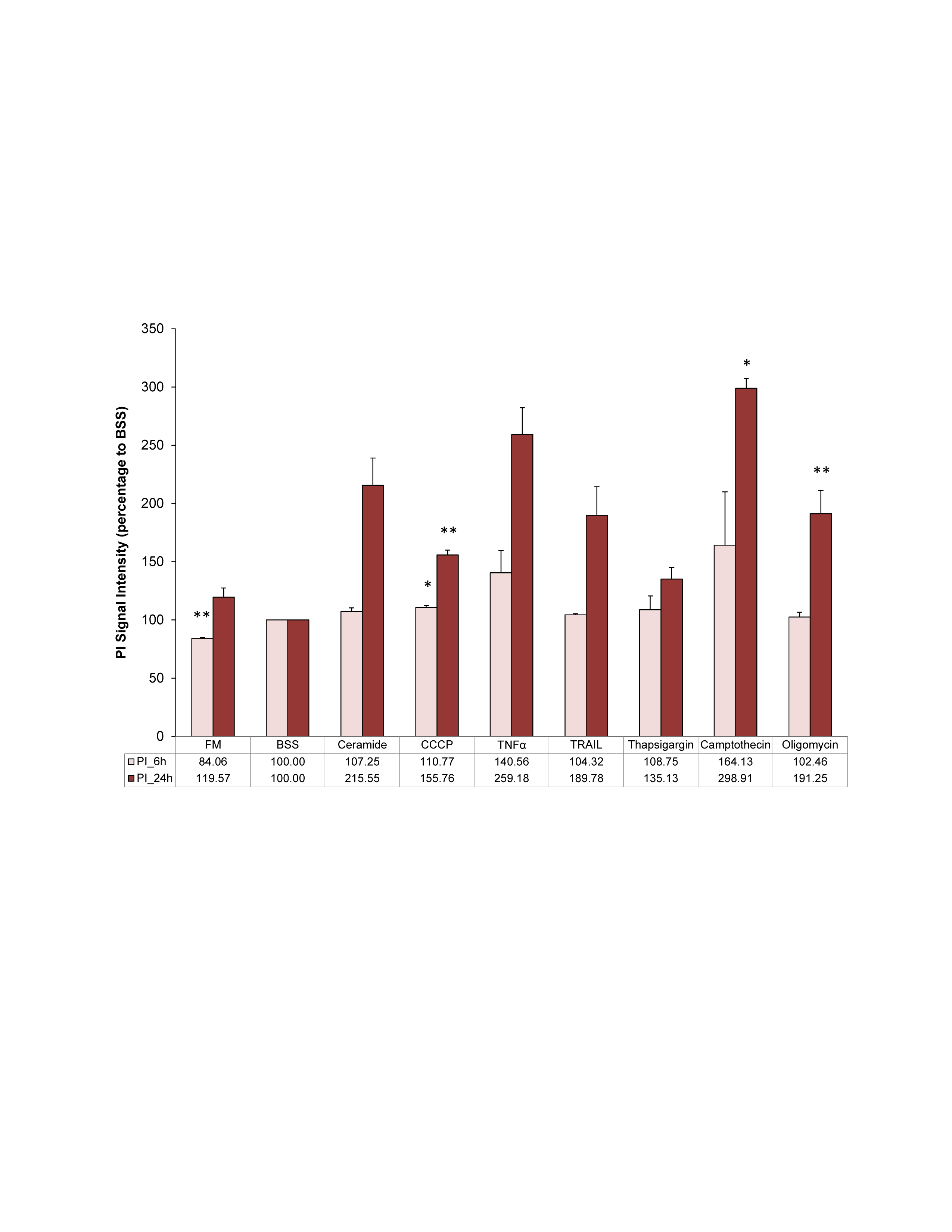

Supplement: Figure S4 — Cell death dataset. Cells were plated in 96 well plates, and cell death was quantified for each condition at 6 hours and 24 hours incubation with indicated drugs at 37°C. Dead cells were stained with propidium iodide (PI, 1.0 ug/ml) and signal intensity measured by plate reader (excitation: 530 nm; emission: 620 nm). Results are normalized to control and represented as percentage ± s.e.m (BSS, 100%). (N = 4). (TIF) [file pone.0028694.s004.tif]

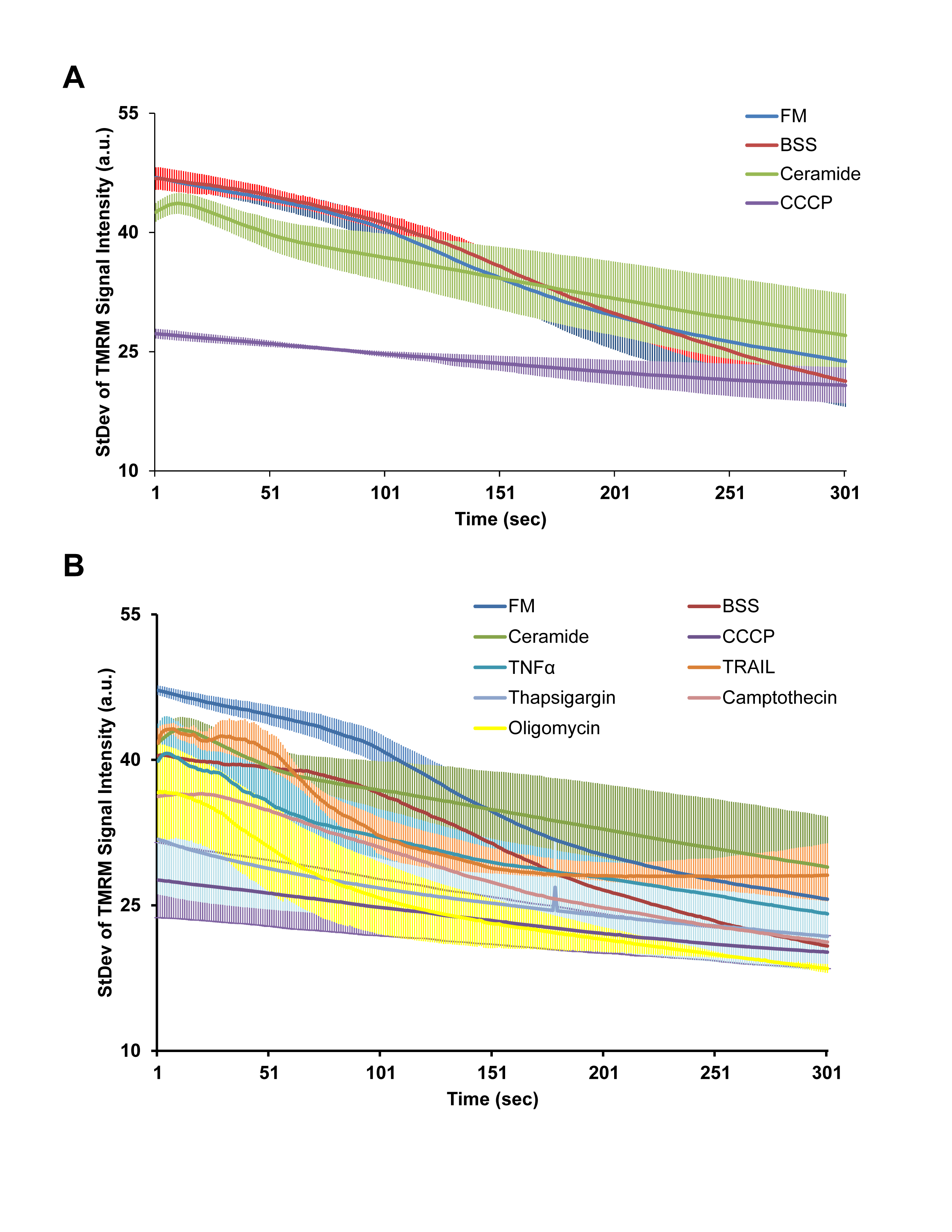

Supplement: Figure S5 — Quantification of ΔΨm sensitivity in response to apoptotic stimuli. MCF-7 wild-type (wt) cells were incubated with tetramethyl rhodamine methyl-ester (TMRM, 25 nM) for 25 minutes at 37°C after 6 hour treatment with the apoptotic drugs. Sequential images of TMRM fluorescence were then acquired every second using exposure times of 20 milliseconds, during a total of 5 minutes. TMRM signal over time is reported as the StDev value, which corresponds to the standard deviation of the average gray values within each individual cell. A) Depolarization profiles of initial conditions. TMRM signal StDev over 5 minutes (301 seconds) for the initial conditions used to build mitochondrial morphology training sets: FM, ceramide (300 µM) and CCCP (20 µM). B) Depolarization profiles of drug selection.- TMRM signal StDev over 5 minutes (301 seconds) for apoptotic conditions: BSS, TNFα (43 ng/mL), TRAIL (20 ng/mL), thapsigargin (1 µM), camptothecin (2 µM) and oligomycin (10 µM). Values are presented as mean ± s.e.m. (N = 4, approx. 400 cells/condition). (TIF) [file pone.0028694.s005.tif]
