# Supplementary material for: Multi-Parametric Analysis and Modeling of Relationships between Mitochondrial Morphology and Apoptosis
Source: PLoS One. 2012 Jan 17;7(1):e28694. doi: 10.1371/journal.pone.0028694 (PMC3260148; doi:10.1371/journal.pone.0028694)
Supplement: Table S1 — List of Features extracted per cell and related to the nucleus. (DOCX) [file pone.0028694.s006.docx]

**Table S1. List of Features extracted per cell and related to the nucleus.**

| C_Texture_AngularSecondMoment_N_3 |
| --- |
| C_Texture_Contrast_N_3 |
| C_Texture_Correlation_N_3 |
| C_Texture_Variance_N_3 |
| C_Texture_InverseDifferenceMom_N_3 |
| C_Texture_SumAverage_N_3 |
| C_Texture_SumVariance_N_3 |
| C_Texture_SumEntropy_N_3 |
| C_Texture_Entropy_N_3 |
| C_Texture_DifferenceVariance_N_3 |
| C_Texture_DifferenceEntropy_N_3 |
| C_Texture_InfoMeas1_N_3 |
| C_Texture_InfoMeas2_N_3 |
| C_Texture_GaborX_N_3 |
| C_Texture_GaborY_N_3 |

C- Cell. N-Nuclei.
