# Supplementary material for: Multi-Parametric Analysis and Modeling of Relationships between Mitochondrial Morphology and Apoptosis
Source: PLoS One. 2012 Jan 17;7(1):e28694. doi: 10.1371/journal.pone.0028694 (PMC3260148; doi:10.1371/journal.pone.0028694)
Supplement: Table S2 — List of Features extracted per cell and related to the cell. (DOCX) [file pone.0028694.s007.docx]

**Table S2. List of Features extracted per cell and related to the cell.**

| C_Children_M_Count |
| --- |
| Means_M_per_C_Location_Center_X |
| Means_M_per_C_Location_Center_Y |
| Means_M_per_C_AreaShape_Area |
| Means_M_per_C_AreaShape_Eccentricity |
| Means_M_per_C_AreaShape_Solidity |
| Means_M_per_C_AreaShape_Extent |
| Means_M_per_C_AreaShape_EulerNumber |
| Means_M_per_C_AreaShape_Perimeter |
| Means_M_per_C_AreaShape_FormFactor |
| Means_M_per_C_AreaShape_MajorAxisLength |
| Means_M_per_C_AreaShape_MinorAxisLength |
| Means_M_per_C_AreaShape_Orientation |

C- Cell. M- Mitochondria.
