# Supplementary material for: Multi-Parametric Analysis and Modeling of Relationships between Mitochondrial Morphology and Apoptosis
Source: PLoS One. 2012 Jan 17;7(1):e28694. doi: 10.1371/journal.pone.0028694 (PMC3260148; doi:10.1371/journal.pone.0028694)
Supplement: Table S3 — List of Features extracted per cell and related to each mitochondrion. (DOCX) [file pone.0028694.s008.docx]

**Table S3. List of Features extracted per cell and related to each mitochondrion.**

| Means_M_per_C_Zernike_0_0 |
| --- |
| Means_M_per_C_Zernike_1_1 |
| Means_M_per_C_Zernike_2_0 |
| Means_M_per_C_Zernike_2_2 |
| Means_M_per_C_Zernike_3_1 |
| Means_M_per_C_Zernike_3_3 |
| Means_M_per_C_Zernike_4_0 |
| Means_M_per_C_Zernike_4_2 |
| Means_M_per_C_Zernike_4_4 |
| Means_M_per_C_Zernike_5_1 |
| Means_M_per_C_Zernike_5_3 |
| Means_M_per_C_Zernike_5_5 |
| Means_M_per_C_Zernike_6_0 |
| Means_M_per_C_Zernike_6_2 |
| Means_M_per_C_Zernike_6_4 |
| Means_M_per_C_Zernike_6_6 |
| Means_M_per_C_Zernike_7_1 |
| Means_M_per_C_Zernike_7_3 |
| Means_M_per_C_Zernike_7_5 |
| Means_M_per_C_Zernike_7_7 |
| Means_M_per_C_Zernike_8_0 |
| Means_M_per_C_Zernike_8_2 |
| Means_M_per_C_Zernike_8_4 |
| Means_M_per_C_Zernike_8_6 |
| Means_M_per_C_Zernike_8_8 |
| Means_M_per_C_Zernike_9_1 |
| Means_M_per_C_Zernike_9_3 |
| Means_M_per_C_Zernike_9_5 |
| Means_M_per_C_Zernike_9_7 |
| Means_M_per_C_Zernike_9_9 |
| Means_M_per_C_Neighbors_NumberOfNeighbors_15 |
| Means_M_per_C_Neighbors_PercentTouching_15 |
| Means_M_per_C_Neighbors_FirstClosestObjectNu_15 |
| Means_M_per_C_Neighbors_FirstClosestXVector_15 |
| Means_M_per_C_Neighbors_FirstClosestYVector_15 |
| Means_M_per_C_Neighbors_SecondClosestObjectN_15 |
| Means_M_per_C_Neighbors_SecondClosestXVector_15 |
| Means_M_per_C_Neighbors_SecondClosestYVector_15 |
| Means_M_per_C_Neighbors_AngleBetweenNeighbor_15 |
| Means_M_per_C_Parent_C |
| Means_M_per_C_Distance_C |

M- Mitochondria. C- Cell.
