# Supplementary material for: Multi-Parametric Analysis and Modeling of Relationships between Mitochondrial Morphology and Apoptosis
Source: PLoS One. 2012 Jan 17;7(1):e28694. doi: 10.1371/journal.pone.0028694 (PMC3260148; doi:10.1371/journal.pone.0028694)
Supplement: Table S4 — Intercellular variances of mitochondrial class subpopulations under the different drug treatments. (DOCX) [file pone.0028694.s009.docx]

**Table S4. Intercellular variances of mitochondrial class subpopulations under the different drug treatments.**

| **StDev (%)** | **Networked** | **Fragmented** | **Swollen** | **Mean**  **StDev (%)** |
| --- | --- | --- | --- | --- |
| **FM** | 31.26 | 22.73 | 14.34 | 22.78 |
| **BSS** | 28.16 | 23.40 | 11.52 | 21.03 |
| **Ceramide** | 18.39 | 18.28 | 18.71 | 18.46 |
| **CCCP** | 7.60 | 16.27 | 21.07 | 14.98 |
| **TNFα** | 29.48 | 24.42 | 18.67 | 24.19 |
| **TRAIL** | 29.31 | 24.67 | 11.09 | 21.69 |
| **Thapsigargin** | 27.21 | 23.75 | 10.67 | 20.54 |
| **Camptothecin** | 31.06 | 21.15 | 27.49 | 26.57 |
| **Oligomycin** | 12.25 | 19.06 | 25.35 | 18.89 |

StDev- Standard Deviation calculated from whole mitochondrial population per condition.
