# Supplementary material for: Multi-Parametric Analysis and Modeling of Relationships between Mitochondrial Morphology and Apoptosis
Source: PLoS One. 2012 Jan 17;7(1):e28694. doi: 10.1371/journal.pone.0028694 (PMC3260148; doi:10.1371/journal.pone.0028694)
Supplement: Information S1 — (DOC) [file pone.0028694.s010.doc]

SUPPORTING INFORMATION

**CellProfiler analysis pipeline**

CellProfiler (CP) modules pipeline used for full image analysis. Note that these steps were tested and optimized for human MCF-7 breast cancer cells stably expressing mito-GFP, and imaged with an epi-fluorescence based Deltavision deconvolution microscope, using a 63x oil-immersion objective. Application of this pipeline to datasets derived using different cell types and imaging platforms may require pipeline adjustment.

CP MODULES:

1. Load Images

2. Rescale Intensity (nuclei)

3. Rescale Intensity (mitochondria)

4. Apply Threshold (mitochondria)

5. Correct Illumination_Calculate (nuclei)

6. Correct Illumination_Apply (nuclei)

7. Smooth or Enhance (mitochondria)

8. Identify Prim Automatic (nuclei)

9. Identify Secondary (cells)

10. Convert to Image (cells)

11. Save Images (cells)

12. Identify Prim Automatic (mitochondria)

13. Convert to Image (mitochondria)

14. Save Images (mitochondria)

15. Measure Object Area Shape (mitochondria)

16. Measure Object Neighbours (mitochondria)

17. Measure Texture (cell)

18. Relate (mitochondria to the cell)

19. Export to Excel

20. Export to Database

1. Load Images:

Deconvolved image stacks were loaded into CellProfiler. The module assigns the respective acquisition channels to each image.

2. and 3. Rescale Intensity:

We applied rescaling method E on individual images by entering a minimum and a maximum intensity value. Pixels range were kept between (0;1), while the highest value and the lowest value of the rescaled image were adjusted to: ´Nuclei´ (0;0.6) and for ´Mitochondria´ image (0.009;1.67).

4. Apply Threshold:

In order to exclude clearly define the boundaries of mitochondria, we applied a binary threshold to the ´mitochondria´ image (0.28). All other setting were kept at standard settings and the original intensity values were retained.

5. Correct Illumination_Calculate:

Here the background intensity was calculated per each image by setting a pixel box size of 150 pixels to the ´nuclei´ images.

6. Correct Illumination_Apply:

The previous calculated background image was subtracted from the image after module 2 (´rescaled nuclei´).

7. Smooth or Enhance:

To derive the cytoplasmic region from the ´mitochondria´ channel we applied a Gaussian Filter smoothing method on mitochondria images (50 pixels box).

8. Identify Primary Automatic:

This module identifies primary objects (nuclei) in the grayscale images obtained from module 6. To identify and segment the nuclei, we chose to a) discard the objects outside the diameter range (100; 300) pixels; b) to merge small objects that are close to larger ones, as they are likely to belong to the nucleus and were mistakenly separated; c) to discard objects (nuclei) touching the image border because part of its cell will be out of the image. We applied Otsu Adaptative for segmentation with a correction factor of 1.2. In order to distinguish and divide clumped objects we selected the Intensity feature and decided on a second step to have filled holes in identified objects,

9. Identify Secondary:

We applied CP Propagation method based on the mitochondria smoothed images from module 7 to identify the cell borders,. (0.05 regularization factor) on an Otsu Adaptative threshold (no correction and full range).

12. Identify Prim Automatic:

Individual mitochondria are segmented by applying the CP Otsu Adaptative (1.2 correction factor). In this case, we have only discarded object lying outside the diameter range (5; 300).

15. Measure Object Area and Shape

This module extracts area and shape features of each individual object (mitochondria) of images from module 14.

16. Measure Object Neighbors:

We considered all object within 15 pixels distance to be neighbors.

17. Measure Texture:

This module extracts texture features of each individual object (cell). We chose a scale of texture of 3.

18. Relate objects:

This module allows for the association of “children” objects (mitochondria) with “parents” objects (cell). In this case, we can quantify how many mitochondria are present per cell as well as obtain a mean value for all measures preformed above averaged per cell.

20. Export to Database:

Measurements described above (modules 15 and 16) are exported to SQL. Data is given for all segmented objects (nuclei, cell and mitochondria) containing their related features. Mean mitochondria features per cell are used for classification.

Full list of features are presented in Table S1 to S3.

**Immunostaining for Cytochrome *c* and COXIV**

MCF-7 cells stably expressing GFP-Bax were grown overnight in 8-well microscopy chambers (ibidi) and subjected to the indicated treatments for 6 hours at 37°C. Cells were fixed with 4% paraformaldehyde (Electron Microscopy Sciences) in phosphate-buffered saline (PBS), permeabilized with 0.3% Triton X-100/PBS, and blocked with 3% BSA. Next, cells were incubated with antibodies against cytochrome *c* (BD Pharmingen) and COXIV (Cell Signalling) overnight at 4°C. After washing, the cells were incubated with Alexa Fluor 546 (Invitrogen) and Alexa Fluor 647 to visualize cytochrome *c* and COXIV, respectively, for 1 hour at room temperature. Cells were imaged by at 63x resolution Objective (approx. 60 cells per condition).

**Cell Death Assay**

MCF-7 wt cells were grown in 96-well plates to approximately 80% confluency. Cells were treated with the drugs for 6 hours or for 24 hours at 37°C, as indicated, and stained with the membrane exclusion dye propidium iodide (PI, 1.0 ug/ml, Invitrogen) for 30 minutes at 37°C. Fluorescence was measured with a Tecan plate reader in bottom-read mode using Magellan V 6.3 software (excitation: 530 nm; emission: 620 nm) and according to manufacturer’s instructions.

.

**Fuzzy Logic Modeling Pipeline**

Fuzzy logic toolbox (Matlab R2009a) was used to perform data integration, with the aim of analyzing non-linear relationships between morphological and functional mitochondrial activities.

Model assembly to explore the relationship between multiple events and a single functional feature was performed according to the following:

1. Data Processing

Raw data from the various experimental procedures was scaled to each maximal observed value in order to make datasets comparable.

2. Assembly of a Sugeno FLS

The fuzzy logic Toolbox was used to generate a fuzzy logic system (FLS) automatically with the existing function genfis1, thereby creating a grid of rules to relate one input MF to exclusively one output membership Function (MF). 2 gaussian functions were used to fuzzify one input. In genfis1, the number of rules is ***m to the power of n***, where ***m*** is the number of input MFs and ***n*** is the number of inputs; hence in our single-input case the number of rules was 2 per model. Upon exploration of the MF parameter space we observed slightly better performance using other non-linear functions. However, the above-mentioned setting was used due to its smaller number of parameters. Linear MFs were chosen as output with standard settings previous to training. Genfis1 requires the experimental data as a matrix. In the case of multiple events and a single functional feature, all columns in the matrix except the last one are considered causative events and the last column is accepted as functional feature.

3. Training of the FLS

Once generated, the FLS was trained using adaptative neuro-fuzzy inference systems (ANFIS). As a learning algorithm, a hybrid of backpropagation coupled to least-squares was employed.

4. Simulation of the FLS

Simulink, the simulation platform coupled to Matlab, was used to create a simulink model to simulate the trained FLS. This model was then run for each experimental datapoint to estimate the value of the desired phenotype. The resulting estimated values were saved in an excel sheet together with the experimental dataset, which were used to calculate the root mean squared error (RMSE).

5. Exhaustive search

The process stated in steps 1, 2 and 3 was then iterated to create the FLS for a total of 30 models representing all potential interactions: Each morphology class influencing a MPT parameter (9 models), the mirror models (9 models), Bax activation influencing each one of the three morphological classes (3 models) and the mirror models (3 models) as well as Bax influencing each MPT parameter (3 models) and the mirror models (3 models).

6. Model selection

For follow-up analysis of the least-error models, see results.
